# Enzyme Inhibitor Studies Reveal Complex Control of Methyl-D-Erythritol 4-Phosphate (MEP) Pathway Enzyme Expression in *Catharanthus roseus*

**DOI:** 10.1371/journal.pone.0062467

**Published:** 2013-05-01

**Authors:** Mei Han, Simon C. Heppel, Tao Su, Jochen Bogs, Yuangang Zu, Zhigang An, Thomas Rausch

**Affiliations:** 1 Centre for Organismal Studies (COS) Heidelberg, Heidelberg University, Heidelberg, Germany; 2 Dienstleistungszentrum Ländlicher Raum-Rheinpfalz, Neustadt, Germany; 3 Northeast Forestry University, Key Laboratory of Forest Plant Ecology, Ministry of Education, Harbin, PR China; Univ. Georgia, United States of America

## Abstract

In *Catharanthus roseus*, the monoterpene moiety exerts a strong flux control for monoterpene indole alkaloid (MIA) formation. Monoterpene synthesis depends on the methyl-D-erythritol 4-phosphate (MEP) pathway. Here, we have explored the regulation of this pathway in response to developmental and environmental cues and in response to specific enzyme inhibitors. For the MEP pathway entry enzyme 1-deoxy-D-xylulose 5-phosphate synthase (DXS), a new (type I) DXS isoform, CrDXS1, has been cloned, which, in contrast to previous reports on type II CrDXS, was not transcriptionally activated by the transcription factor ORCA3. Regulation of the MEP pathway in response to metabolic perturbations has been explored using the enzyme inhibitors clomazone (precursor of 5-ketochlomazone, inhibitor of DXS) and fosmidomycin (inhibitor of deoxyxylulose 5-phosphate reductoisomerase (DXR)), respectively. Young leaves of non-flowering plants were exposed to both inhibitors, adopting a non-invasive *in vivo* technique. Transcripts and proteins of DXS (3 isoforms), DXR, and hydroxymethylbutenyl diphosphate synthase (HDS) were monitored, and protein stability was followed in isolated chloroplasts. Transcripts for *DXS1* were repressed by both inhibitors, whereas transcripts for *DXS2A*&*B*, *DXR* and *HDS* increased after clomazone treatment but were barely affected by fosmidomycin treatment. DXS protein accumulated in response to both inhibitors, whereas DXR and HDS proteins were less affected. Fosmidomycin-induced accumulation of DXS protein indicated substantial posttranscriptional regulation. Furthermore, fosmidomycin effectively protected DXR against degradation *in planta* and in isolated chloroplasts. Thus our results suggest that DXR protein stability may be affected by substrate binding. In summary, the present results provide novel insight into the regulation of DXS expression in *C. roseus* in response to MEP-pathway perturbation.

## Introduction

*Catharanthus roseus* (Madagascar periwinkle) produces a variety of pharmacologically active monoterpene indole alkaloids (MIAs), e.g. ajmalicine and serpentine effective as antihypertensive agents, and the dimeric MIA derivatives vinblastine and vincristine used as anti-cancer drugs. Due to its pharmaceutical potential, *C. roseus* has become one of the best-studied medicinal plants with respect to secondary metabolism [1]. However, attempts towards upgrading MIA accumulation have as yet met with moderate success, and continuous efforts are directed to further elucidate the regulation of MIA biosynthesis [2], [3]. MIA biosynthesis involves the condensation of tryptamine (indole moiety) with secologanin (monoterpene-secoiridoid moiety). Secologanin is derived from the basic isoprenoid units isopentenyl diphosphate (IPP) and its isomer dimethylallyl diphosphate (DMAPP), and supply of secologanin is considered to be rate-limiting for MIA biosynthesis [4]–[6]. Thus, isoprenoid precursor flux may impact on secologanin availability for MIA biosynthesis.

**Figure 1:**
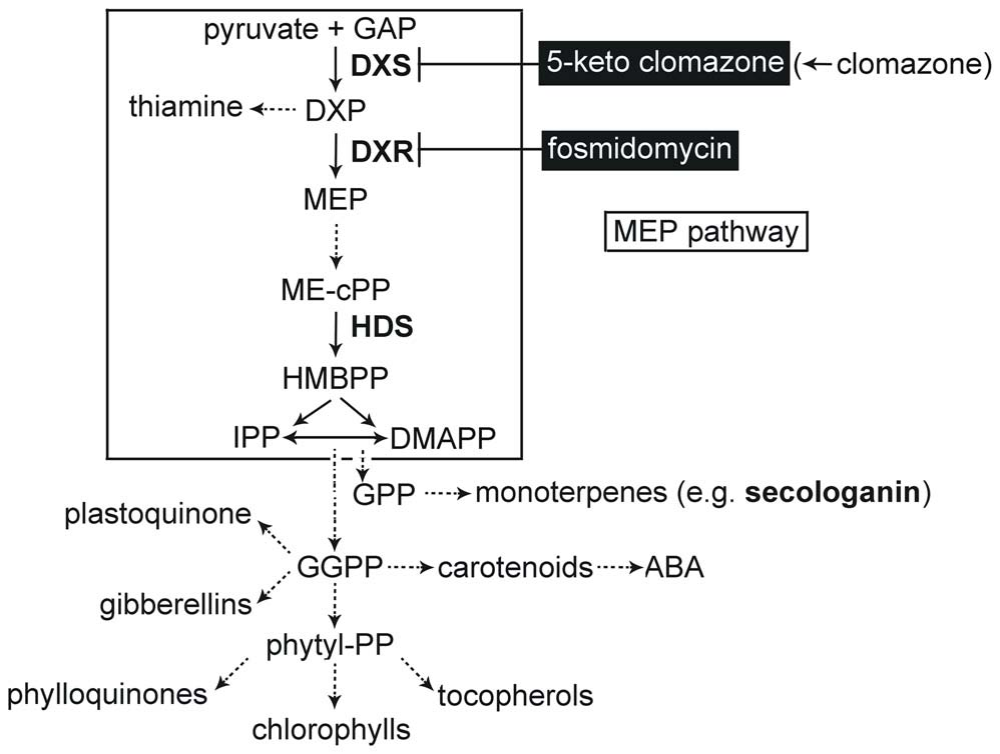
Schematic view of plastidic methylerythritol 4-phosphate (MEP) pathway providing the precursors for secologanin (monoterpene) synthesis. Enzymes analyzed in the present study are in bold face (DXS, 1-deoxy-D-xylulose 5-phosphate synthase; DXR, deoxyxylulose 5-phosphate reductoisomerase; HDS, hydroxymethylbutenyl diphosphate synthase). GAP, glyceraldehyde 3-phosphate; DXP, deoxyxylulose 5-phosphate; MEP, methylerythritol 4-phosphate; MEcPP, methylerythritol 2,4-cyclodiphosphate; HMBPP, hydroxymethylbutenyl diphosphate; IPP, isopentenyl diphosphate; DMAPP, dimethylallyl diphosphate; GPP, geranyl diphosphate; GGPP, geranylgeranyl diphosphate. ABA, abscisic acid. The step branching to thiamine from DXP is indicated. Furthermore, inhibition of DXS and DXR by 5-ketoclomazone (formed *in planta* from clomazone) and fosmidomycin, respectively, is highlighted. Dashed arrows indicate multiple steps.

In higher plants, two pathways are used for the synthesis of the basic isoprenoid units, i.e. the cytosolic mevalonate (MVA) pathway generating precursors for sesqui- (C15) and triterpenes (C30), such as phytosterols, dolichols, and farnesyl residues for protein prenylation, and the plastidic methyl-D-erythritol 4-phosphate (MEP) pathway (Fig. 1) for the synthesis of carotenoids, plastoquinones, phytol conjugates (such as chlorophylls and tocopherols) and hormones (gibberellins and abscisic acid) [7], [8]. Previous work has confirmed that secondary metabolites, such as MIAs derive their monoterpene moiety from the MEP pathway [9]. The MEP pathway operates in a broad range of organisms, including bacteria, certain protozoa, green algae, and higher plants. Extensive research has elucidated its biosynthetic steps, structure-function relationships of individual enzymes, and its role for terpenoid biosynthesis [7], [10], [11].

The first step of the MEP pathway is catalyzed by 1-deoxy-D-xylulose 5-phosphate synthase (DXS), converting pyruvate and glyceraldehyde-3-phosphate to 1-deoxy-D-xylulose 5-phosphate (DXP, Fig. 1). Previous studies have shown that DXS is highly regulated during plant development and in response to abiotic and biotic stress [12]–[15]. As the expression of DXS is closely correlated with accumulation and decrease of plastid isoprenoids, the DXS enzyme has been considered as a rate-limiting enzyme for MEP pathway flux [7], [16], [17]. In general, the MEP pathway enzymes are encoded by single genes [17]; however, DXS is an exception. In several plant species, DXS is encoded by a small gene family. The DXS-encoding genes cluster into (at least) two clades, the isoforms displaying differential expression patterns. Type I DXS genes are functionally related to the photosynthetic process (i.e. pigment biosynthesis), whereas type II DXS genes appear to be involved in the synthesis of isoprenoid-derived secondary metabolites [17], [18]. The second enzyme in the MEP pathway is 1-deoxy-d-xylulose-5-phosphate reductoisomerase (DXR). Overexpression of DXR stimulates the synthesis of MEP pathway-derived isoprenoids like essential oil [19] and taxadiene (in transgenic *Arabidopsis*; [20]), while reduced expression of DXR resulted in variegation, pigment reduction and arrest of chloroplast development. Thus, besides DXS, the enzyme DXR represents another potential regulatory control point in the MEP pathway. The penultimate step of the MEP pathway is catalyzed by hydroxymethylbutenyl 4-diphosphate synthase (HDS). Its expression is negatively correlated with plant resistance against pathogens [21], and the turnover of HDS appears to be a bottleneck of the MEP pathway in plant in response to oxidative stress [22].

Inhibitors of DXS and DXR have been used as an additional tool to study the regulation of isoprenoid production in plants [15], [20], [23]–[27]. Clomazone (2-[(2-chlorophenyl) methyl]-4,4-dimethyl-3- isoxazolidinone), a soil-applied herbicide [28], is the precursor of 5-ketoclomazone, an inhibitor of DXS [29], [30], and sensitivity of recombinant *C. roseus* DXS to 5-ketoclomazone has been demonstrated [29]. Fosmidomycin (3-(*N-*formyl-*N*-hydroxyamino) propylphos-phonic acid), also known as FR-31564, is a specific inhibitor of DXR [31].

Recently, studies on MEP pathway enzyme expression in *Arabidopsis thaliana* have revealed the existence of posttranscriptional control(s) [23], [25], [26]. Thus, application of fosmidomycin led to an accumulation of DXS protein, apparently without increase in DXS transcript amount [23]. Furthermore, proteomic analysis of an *Arabidopsis* Clp protease mutant revealed increased levels of MEP pathway enzyme proteins [26], [32], suggesting that proteolytic turnover may be involved in fine tuning of MEP pathway enzyme levels. As yet, little is known about the multiple levels of MEP pathway regulation in *C. roseus*. Interestingly, overexpression of DXS in *C. roseus* hairy roots stimulated the accumulation of several MIAs [33], and DXS expression was induced in ORCA3 overexpression *C. roseus* cell lines (ORCA3: a jasmonate-responsive APETALA2 (AP2)-domain transcription factor activating MIA biosynthesis [34], [35]). Besides, various analogues of the DXR inhibitor fosmidomycin inhibited MIA synthesis in *C. roseus* cells [36], [37], indicating that MEP pathway flux may impact on MIA biosynthesis. *In planta*, expression of MEP pathway genes appears to be concentrated in internal phloem associated parenchyma (IPAP) cells [2], [38]. Furthermore, several MEP pathway genes are induced upon stimulation of MIA biosynthesis [39], [40]–[42], However, the regulation of corresponding protein levels remains largely unknown.

Here, the expression of three MEP pathway enzymes in *C. roseus*, namely DXS (three isoforms), DXR, and HDS has been explored by using clomazone and fosmidomycin as tools to perturb MEP pathway flux. In particular, the following questions have been addressed: i) Do DXS isoforms respond differentially to depletion of MEP pathway intermediates (and/or downstream isoprene-derived products)? ii) Does interruption of MEP pathway flux either prior to or after 1-deoxy-D-xylulose 5-phosphate (DXP) result in differential responses for MEP pathway enzyme expression? iii) Do posttranscriptional control and/or protein turnover contribute to DXS enzyme protein accumulation? and iv) Does exposure of MEP pathway target enzymes to specific inhibitors impact on their proteolytic turnover in the chloroplast?

## Results

### cDNA cloning of a novel type I *DXS* isoform of *C. roseus*

Previous work had indicated the existence of two *DXS* genes in *C. roseus* (*CrDXS*, AJ011840; *CrDXS2*, DQ848672). However, phylogenetic analysis revealed that both genes group with the type II clade (Fig. S1). To explore the differential roles of DXS-encoding genes in *C*. *roseus*, we have cloned by RACE approach an additional *DXS* isoform which belongs to the type I clade (Fig. S1). This novel *CrDXS* cDNA, named *CrDXS1* (KC625536), encompasses an ORF of 719 amino acids with a calculated Mr of 77.5 kDa, and includes 258 bp of 5′-UTR and 248 bp of 3′UTR, respectively. For consistency, we have renamed the previously cloned isoforms as *CrDXS2A* (*CrDXS*, AJ011840) and *CrDXS2B* (*CrDXS2*, DQ8486762), respectively. When subjecting *CrDXS1* to PSI-BLAST analysis (http://blast.ncbi.nlm.nih.gov/Blast.cgi), its protein sequence displayed high similarity (i.e. sequence identity of 80–87%) with type I DXS sequences from other plants, while comparison with CrDXS2A and CrDXS2B revealed only 73% identity. Moreover, like all previously cloned plant DXS proteins, CrDXS1 includes an N-terminal transit peptide of 57 amino acids (predicted by the ChloroP program, http://www.cbs.dtu.dk/services/ChloroP/), and the estimated Mr for mature CrDXS1 is 71.2 kDa.

### Expression of DXS isoform mRNAs and DXS protein respond to developmental and stress-related cues

**Figure 2:**
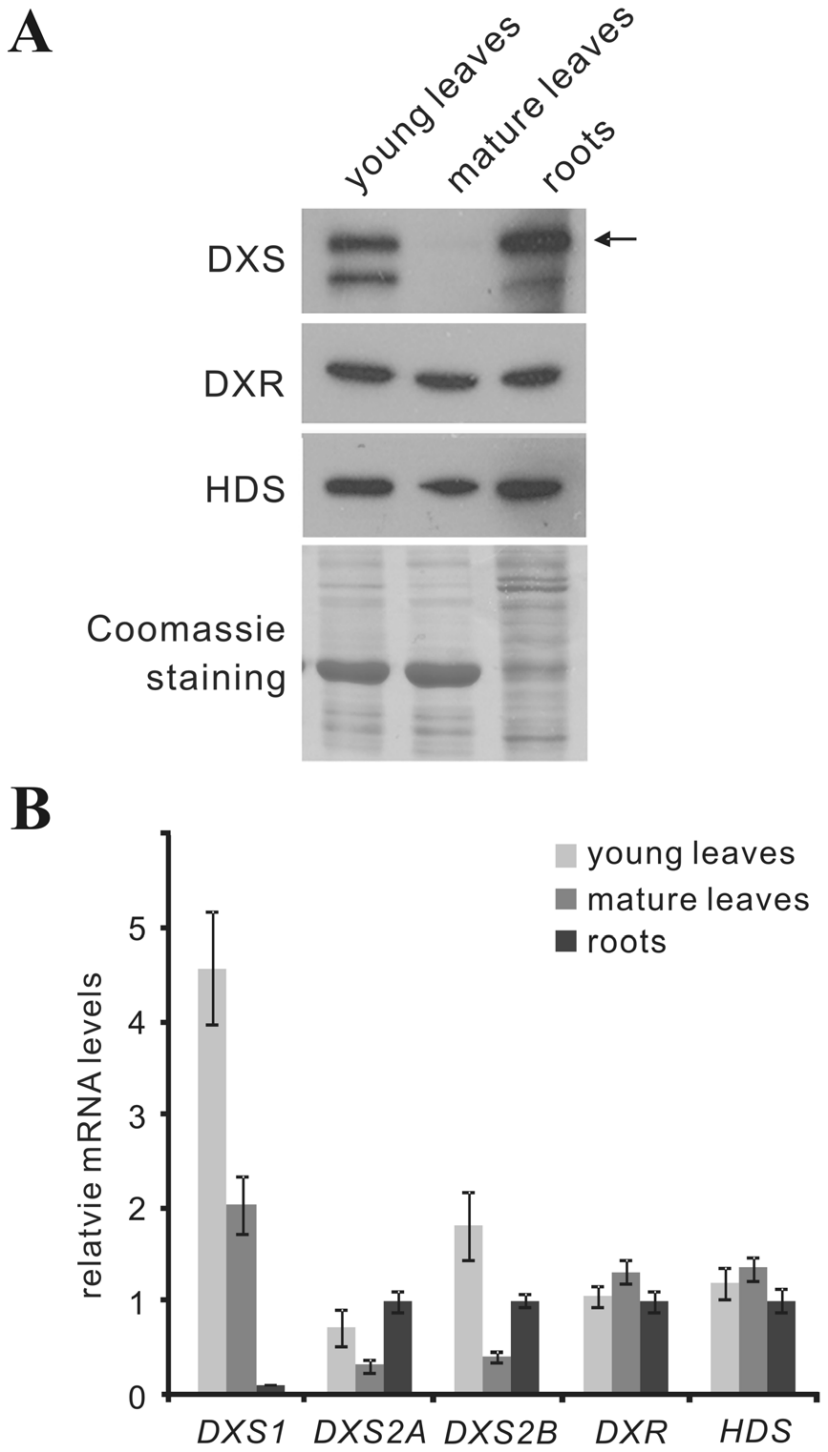
Tissue-specific expression of MEP pathway genes in young leaves, mature leaves and roots of 6-week-old non-flowering *C. roseus* plants, at protein and transcript level, respectively. Soil-grown periwinkle (*Catharanthus roseus*) plants were cultivated in a growth chamber at 25°C with 14 hrs light period (170 µmol m^−2^ s^−1^) and 22°C during dark period. **(A)** DXS, DXR, and HDS proteins were detected by immunoblot with polyclonal antisera raised against recombinant CrDXS2A, DXR and HDS proteins. Equal sample loading was confirmed by Coomassie staining. The arrow marks the position of mature CrDXS2A protein. **(B)** transcript amounts for DXS isoforms (1, 2A & 2B), DXR, and HDS were determined by qPCR (the experiment was performed 3 times, values from a representative experiment are presented ± SD), relative to the geometric mean of multiple reference genes according to Vandesompele et al. [60].

The expression of DXS, DXR and HDS proteins in young leaves, mature (i.e. fully expanded) leaves and roots of 6-week-old non-flowering *C. roseus* plants was determined by immunoblotting (Fig. 2A), using polyclonal antisera raised against recombinant CrDXS2A, CrDXR and CrHDS proteins, respectively. While the latter two antisera are specific for their single gene target enzymes, the antiserum raised against CrDXS2A also detects CrDXS2B and CrDXS1 proteins, respectively, albeit with different intensity (2A≃1>>2B; see Fig. S3); note that the pairwise amino acid sequence identities are: 2A/2B: 74%, 2A/1: 74%, 2B/1: 73%). The corresponding transcripts were quantified by qPCR (Fig. 2B).

The two single gene-encoded MEP pathway enzymes DXR and HDS were expressed to similar degrees in all three organs at protein and transcript level, respectively (Fig. 2). Conversely, DXS protein showed similar abundance in young leaves and roots, but only very weak expression in mature leaves, this being consistent with the corresponding transcript levels of *CrDXS2A&B* (Fig. 2A,B). Note that the CrDXS2A antiserum detects, in addition to the main band at the predicted size of mature DXS2A proteins, a band of higher mobility (apparent Mr 60 kDa); this immune signal is suppressed by preincubation of the antiserum with recombinant CrDXS2A protein, indicative of a CrDXS2A degradation/processing product.

**Figure 3:**
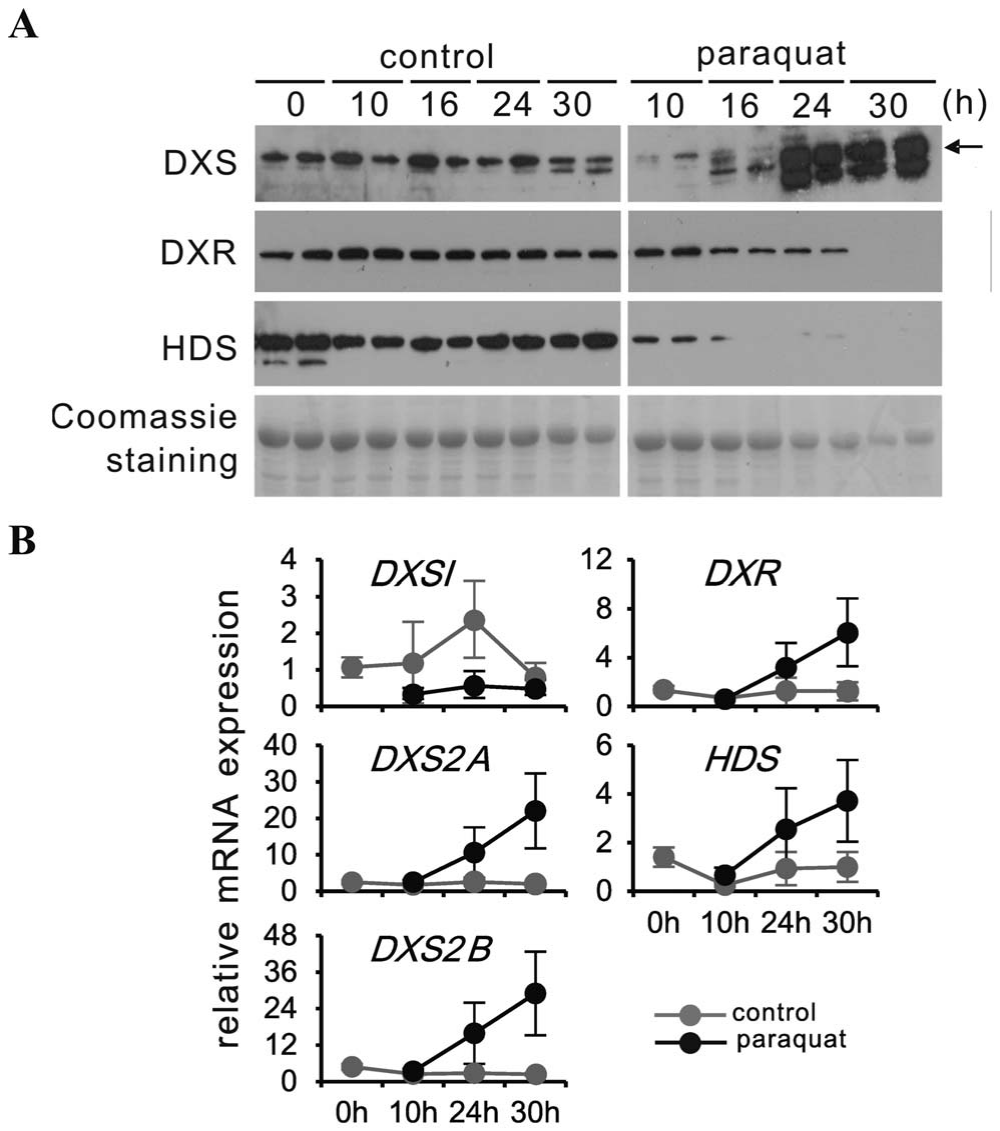
Effect of paraquat treatment on the expression of MEP pathway genes at protein and transcript level, respectively, in leaf discs obtained from mature leaves of 6-week-old *C. roseus* plants. Leaf discs (5 mm diameter) were collected from fully expanded leaves and floated on distilled water in the presence or absence of 0.5 µM paraquat for the indicated time intervals at 25°C under continuous light (270 µmol m^−2^ s^−1^). **(A)** DXS, DXR and HDS proteins were detected by immunoblot. Equal sample loading was confirmed by Coomassie staining. The arrow marks the position of mature CrDXS2A protein.**(B)** transcript amounts for DXS (isoforms 1, 2A & 2B), DXR and HDS were determined by qPCR relative to the geometric mean of multiple reference genes according to Vandesompele et al. [60]. The experiment was performed 3 times; values from a representative experiment are presented ± SD.

To explore the impact of oxidative stress on the expression of MEP-pathway enzymes, leaf discs (from mature leaves) were exposed to a 0.5 µM paraquat (methyl viologen) solution (Fig. 3). Leaf discs of control treatment did not show any bleaching over a period of 30 hrs, whereas paraquat-exposed leaf discs started to bleach after 10 hrs (Fig. S4). In the control treatment, *CrDXS1* transcripts slightly increased during the first 24 hrs, followed by a decline to initial level, whereas in paraquat-treated leaf discs transcripts were expressed at very low level (Fig. 3B). Conversely, *CrDXS2A&B* transcripts remained low in control discs but were strongly induced by paraquat treatment (Fig. 3B). While in control samples, DXS protein amount remained low and fairly unchanged, the paraquat-mediated induction of *CrDXS2A&B* transcripts correlated with a strong increase in DXS protein (Fig. 3A). These results reveal that in response to paraquat treatment, the expression of DXS protein was largely controlled at the transcriptional level, i.e. strong induction of *CrDXS2A&B* mRNAs, accompanied by a loss of *CrDXS1* mRNA. Paraquat-induced oxidative stress also affected protein and transcript levels for DXR and HDS, respectively (Fig. 3A,B). While their transcripts appeared to be co-regulated with *CrDXS2A&B*, DXR and HDS protein levels strongly declined, the protein loss being most pronounced for HDS. In summary, the data support the notion that in mature leaves, *CrDXS1* performs a housekeeping function, whereas *CrDXS2A&B* substitute under conditions of paraquat exposure.

### CrDXS isoforms are differentially regulated by the transcription factor ORCA3

**Figure 4:**
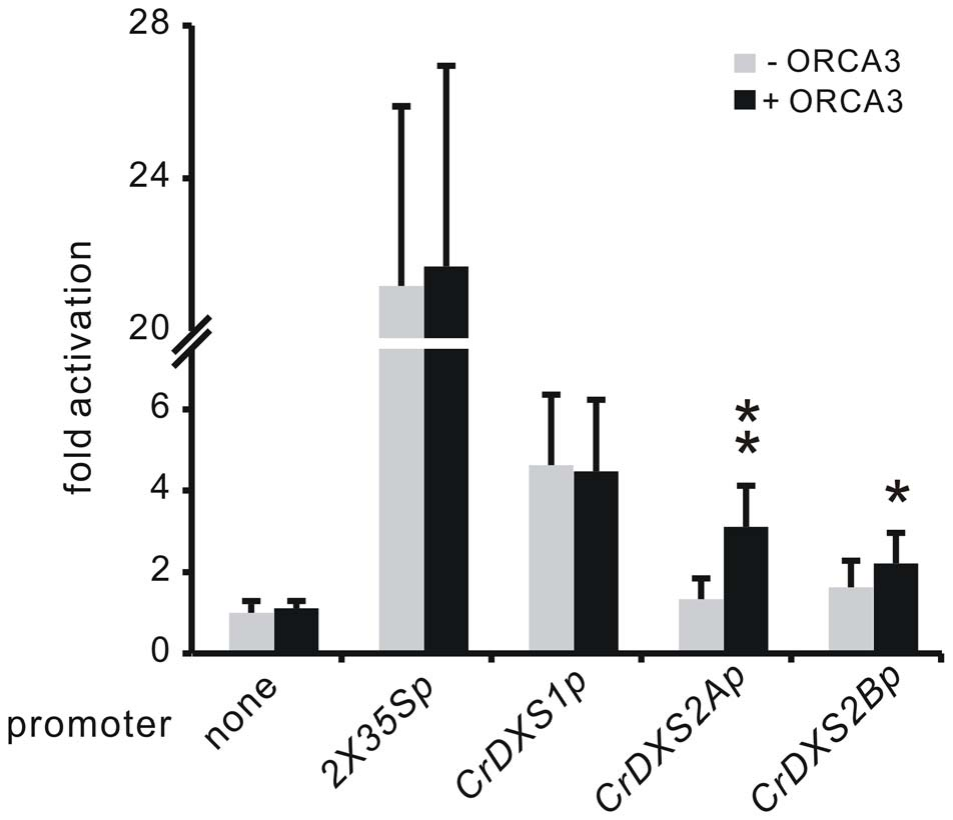
Activation of DXS isoforms' promoters (*CrDXS1p, CrDXS2Ap and CrDXS2Bp*) by the transcription factor ORCA3, analyzed via transient expression of promoter-luciferase fusions in leaves of 6-week old *C. roseus* plants. Results are expressed as fold change of promoter activation, based on four independent assays. The promoter activity was determined by the ratio of luciferase (LUC) activity to renilla expression. Dual *CaMV* 35S promoter was employed as a positive control showing high LUC activity, while promoterless LUC control (pGreenII 0800-LUC vector) reflected the LUC background value. *DXS* promoter sequences were fused to the firefly luciferase and transiently co-transformed with ORCA3 or without (mock control). *Agrobacteria*-mediated transient transformation was carried via leaf infiltration of 6-week-old soil-grown *C. roseus* plants. Two days after transformation, infiltrated leaves were harvested and subjected to dual-luciferase reporter assay. Error bars represent the standard deviation of four independent experiments for promoter activity analysis. The asterisks represent significant difference (** *p*<0.001, * *p*<0.1 by student's t-test).

The differential expression of *CrDXS1* versus *CrDXS2A&B* in different tissues and in response to paraquat exposure indicated distinct mechanisms for their transcriptional regulation. As previous work had shown that in cell culture, *CrDXS2A* is responsive to ORCA3 activation [34], we explored whether *in planta* the DXS isoforms respond differentially to this transcription factor. The promoters of all three *CrDXS* isoforms (including about 2 kb sequences upstream of translation start), were isolated and inserted into the multiple cloning site of pGreenII 0800-LUC vector [43] to drive the expression of firefly luciferase (LUC). After mobilization into *Agrobacterium*, the resulting constructs were co-infiltrated with *Agrobacterium* cells carrying a *CaMV35S*-ORCA3 construct into the first pair of fully expanded mature leaves of 6-week-old soil-grown *C. roseus* plants. Subsequently, promoter activities of DXS isoforms were analyzed by the dual luciferease assay [43]. In the presence of ORCA3, the *DXS2A* promoter activity was strongly (∼3 fold) induced (Fig. 4), and the same was true for *DXS2B*, albeit to a low extent. Conversely, the promoter of *CrDXS1* did not respond to ORCA3. As ORCA3-regulated genes are tightly linked to MIAs accumulation [34], the results suggests that *CrDXS2A&2B*, but not *CrDXS1*, are involved in MIA biosynthesis. The differential affinity of DXS promoters to ORCA3 is consistent with the notion that only type II DXS isoforms contribute to biosynthesis of secondary metabolites [14], [44], [45].

### Clomazone strongly induces DXS protein and a coordinate up-regulation of transcripts for *DXS2A/B*, *DXR* and *HDS*

**Figure 5:**
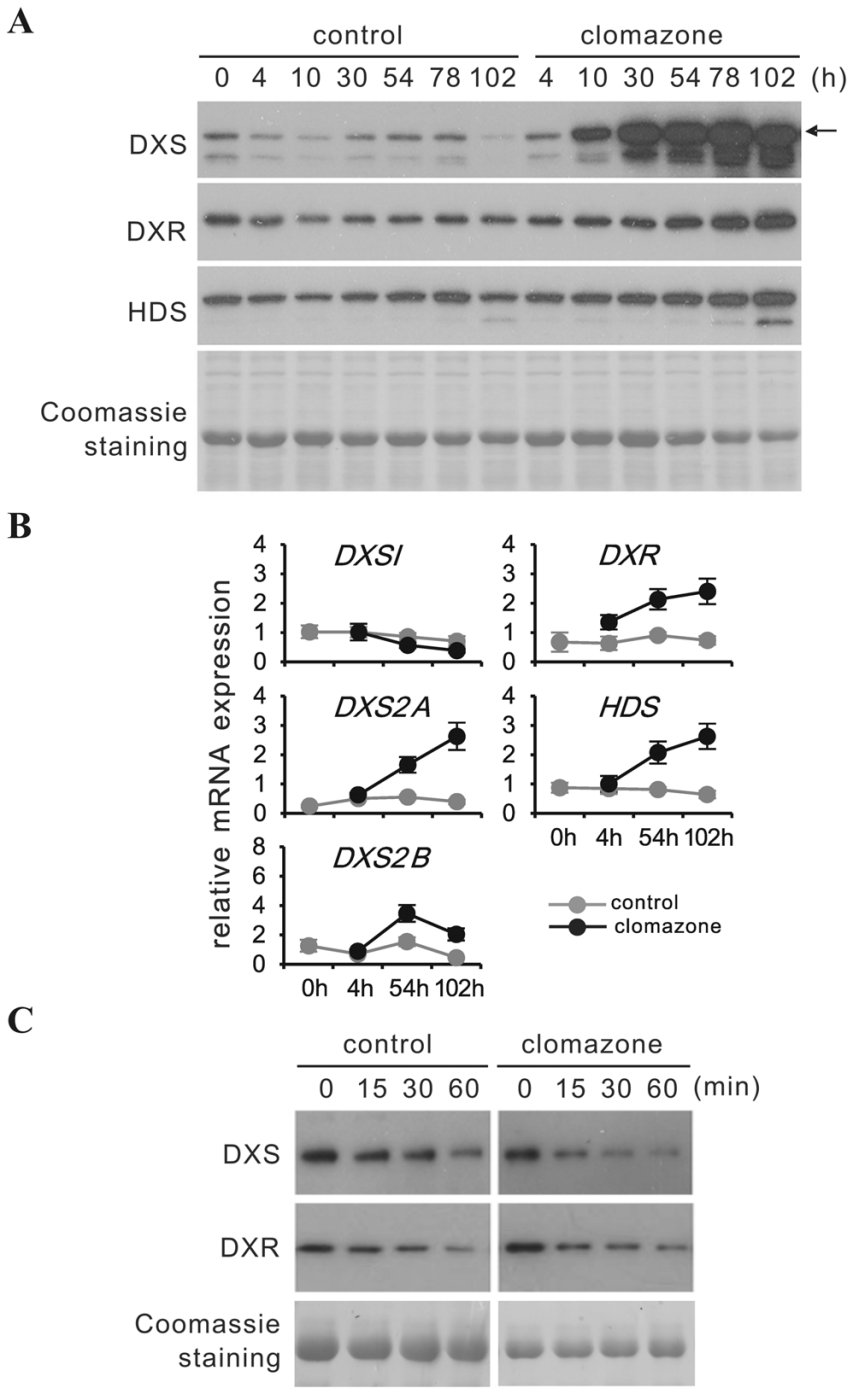
Time course of *in vivo* clomazone treatment on the expression of MEP pathway genes, and subsequent degradation of DXS and DXR proteins in isolated chloroplasts. For each plant, two pairs of mature leaves were injected with a 50 µM clomazone solution (or water for control) until the entire leaf blades were fully soaked, using a 1 ml needleless syringe applied to the lower epidermis. For each time point, young leaves were pooled from three independent plants and processed for MEP pathway protein and transcript analysis, respectively. **(A)** DXS, DXR and HDS proteins were detected by immunoblot. Equal sample loading was confirmed by Coomassie staining. The arrow marks the position of mature CrDXS2A protein. **(B)** transcript amounts for DXS isoforms (1, 2A & 2B), DXR and HDS were determined by qPCR relative to the geometric mean of multiple reference genes according to Vandesompele et al.[60]. The experiment was performed 3 times, values from a representative experiment are presented ± SD. **(C)** chloroplasts were isolated from young leaves of 6-week-old soil-grown *C. rosues* control and 50 µM clomazone-treated plants (see **(A)** this Figure) 78 hours after treatment. Chloroplasts were incubated for 1 h in the light (100 µmol m^−2^ s^−1^) at 25°C in the presence of 5 mM ATP. Aliquots were taken at 0, 15, 30 and 60 minutes and used for protein extraction. DXS and DXR proteins were detected by immunoblot. Note that to obtain similar signal intensity at time point 0, the loading amount of protein from control samples (chloroplast isolated from water infiltrated plants) was twice that of clomazone samples.

To explore the response of MEP pathway enzyme expression to a block of the entire pathway, we have monitored the effects of an enzyme inhibitor for DXS. Clomazone (50 µM, aqueous solution) was applied to the first two pairs of fully expanded mature leaves of 6-week-old non-flowering *C. roseus* plants (Fig. S5) via injection with a needleless plastic syringe, thereby flooding the entire intracellular space with clomazone solution. Tissue samples from young leaves were taken at 0 to 102 hrs after clomazone application and analyzed for MEP pathway enzyme expression at transcript and protein level, respectively (Fig. 5A,B). In emerging young leaves, bleaching was observed as early as 30 hrs after clomazone injection (not shown), and after 78 hrs extended areas of the expanding leaves were strongly bleached (Fig. S5), indicating rapid systemic spreading of the compound. Conversely, fully expanded leaves did not show any bleaching during the chosen time interval. As even the injected (fully expanded) leaves showed little bleaching over time, it is concluded that in mature leaves pigment turnover is too slow to reveal inhibition of MEP pathway flux, in marked contrast to young growing leaves, where *de novo* synthesis of leaf pigments is blocked. However, it cannot be excluded that transport efficiencies for clomazone may differ according to the developmental stage of the leaf.

The effect of clomazone on MEP pathway enzyme expression in young leaves (Fig. 5A) revealed a dramatic increase in DXS protein accumulation (Fig. 5A), starting as early as 10 hours after clomazone application. At transcript level, *CrDXS1* expression slightly declined, whereas *CrDXS2A&B* showed a moderate increase, paralleled by comparable up-regulation of DXR and HDS transcripts (Fig. 5B); however, DXR and HDS protein levels showed only a moderate increase. To confirm that clomazone treatment induces DXS protein to a similar degree as the active derivative 5-ketoclomazone, a similar experiment was performed with 5-ketoclomazone (Fig. S7). The effect of a treatment with 50 µM 5-ketoclomazone on DXS protein accumulation was slightly less pronounced than observed for clomazone, although 5-ketoclomazone caused more bleaching.

To address possible effects of clomazone treatment on DXS protein turnover, the time course of DXS protein decline in isolated chloroplasts from control and clomazone-treated plants was monitored. Three days after clomazone treatment, chloroplasts were isolated and incubated at 25°C for up to 60 min and aliquots were analyzed for DXS protein content (Fig. 5C). In chloroplasts from clomazone-treated leaves, the time-dependent decline of DXS protein was even faster than in chloroplasts from control plants, excluding an increased stability of DXS protein in clomazone-treated leaves as a cause for DXS protein accumulation. Note, that loaded sample amount and film exposure time for signal detection of clomazone-treated samples were adjusted to obtain the same initial immune signal strength as for the control sample at 0 min.

In summary, blocking MEP pathway flux by inhibition of its entry enzyme caused a massive accumulation of DXS protein, correlating with a decline of *CrDXS1* mRNA and an increase of *CrDXS2A* (and to a lesser degree *CrDXS2B*) transcripts. The strong increase of DXS protein in response to clomazone treatment may result from transcriptional up-regulation of *CrDXS2A&B* expression and/or an increased translation efficiency forthe existing mRNAs, as observed in *Arabidopsis* [46].

### Fosmidomycin, a specific inhibitor of DXR, differentially affects expression of transcript levels of DXS isoforms and strongly induces DXS protein

**Figure 6:**
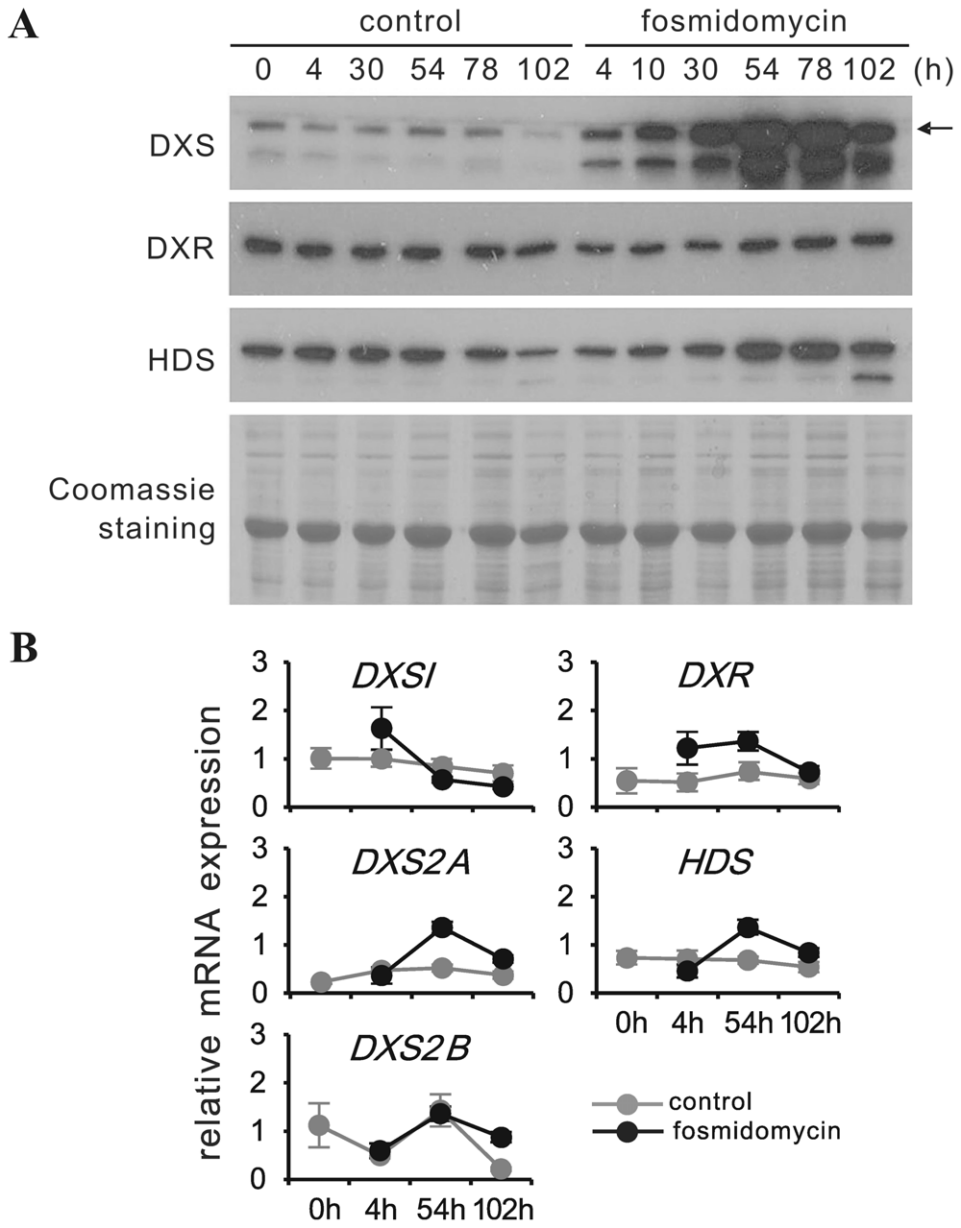
Time course of *in vivo* fosmidomycin treatment on the expression of MEP pathway genes in young leaves of 6-week-old *C. roseus* plants at protein and transcript level, respectively. For each plant, two pairs of mature leaves were injected with a 50 µM fosmidomycin solution. Application procedure and time course of sampling was as described for clomazone treatment (see Figure 5). **(A)** DXS, DXR and HDS proteins were detected by immunoblot. Equal sample loading was confirmed by Coomassie staining. The arrow marks the position of mature CrDXS2A protein. **(B)**, transcript amounts for DXS isoforms (1, 2A & 2B), DXR, and HDS were determined by qPCR, relative to the geometric mean of multiple reference genes according to Vandesompele et al. [60]. The experiment was performed 3 times, values from a representative experiment are presented ± SD.

To block MEP pathway flux at the level of DXR enzyme activity, i.e. allowing the formation of 1-deoxy-D-xylulose 5-phosphate (DXP) while blocking the remaining MEP pathway, a 50 µM aqueous solution of fosmidomycin was applied to the first two pairs of fully expanded mature leaves of 6-week-old *C. roseus* plants as described above. Again, tissue samples from young leaves were collected at 0 to 102 hrs after inhibitor application and analyzed for MEP pathway enzyme expression at protein and transcript level, respectively (Fig. 6A,B). Similar to the results obtained with clomazone application, fosmidomycin caused a dramatic accumulation of DXS protein (Fig. 6A), while expression of *CrDXS1* mRNA declined (Fig. 6B). However, in contrast to clomazone treatment, inhibition of DXR had only a modest effect on the expression of *CrDXS2A&B* mRNAs. Likewise, transcript amounts for MEP pathway enzymes DXR and HDS were only weakly and transiently affected, showing an increase at 54 hrs after fosmidomycin application and declining thereafter (Fig. 6B).

**Figure 7:**
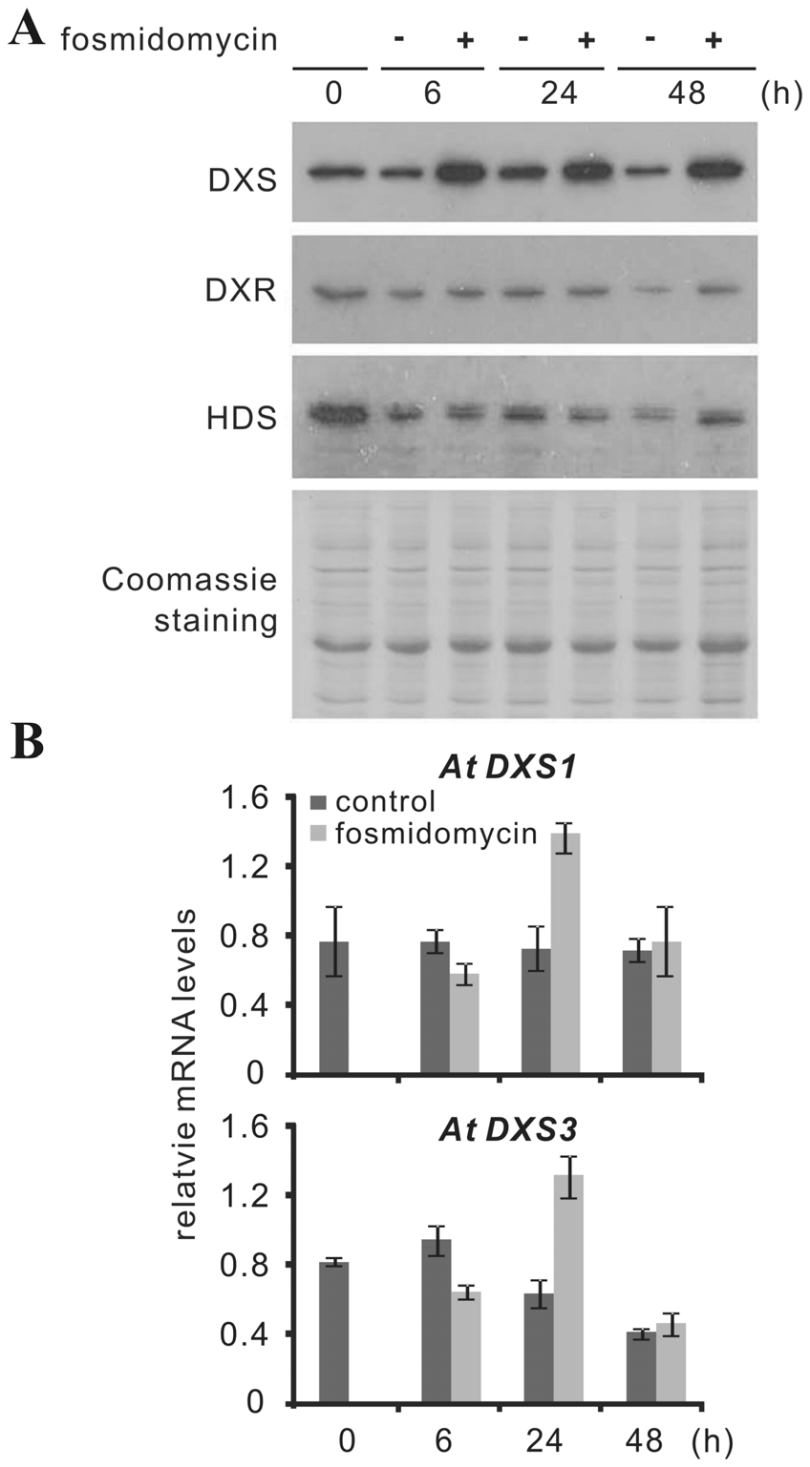
Effect of fosmidomycin on the expression of DXS protein and DXS isoform (1, 3) transcripts in 8-day-old seedlings of *Arabidopsis thaliana.* *A. thaliana* Ler seedlings were cultivated for 8 days at 22°C in petri dishes on filter paper as described by Guevara-Garcia et al. [23]. Light period was 16 hrs (230 µmol m^−2^ s^−1^). Thereafter, 5 ml of a 100 µM fosmidomycin solution (or water as control) were added. After the indicated time intervals, seedlings were harvested and processed for MEP pathway protein and transcript analysis, respectively. **(A)** DXS, DXR, and HDS proteins were detected by immunoblot with polyclonal antisera raised against recombinant *C. roseus* DXS2A, DXR and HDS proteins. Note that exposure time was five times longer for Arabidopsis samples than for *C. roseus* samples. **(B)** transcript amounts for DXS isoforms (1& 3) were determined by qPCR. Relative transcript levels were calculated by normalizing to actin as reference gene. Means of triplicate values from a representative experiment are presented ± SD.

In a previous study with *Arabidopsis* seedlings, an induction of DXS protein after fosmidomycin treatment was observed without any increase of DXS transcript [23]. As this study did not monitor possible differences between DXS isoforms, we have repeated this experiment by individually monitoring the transcript levels of the two expressed DXS isoforms *AtDXS1* and *AtDXS3* in 8-day-old *Arabidopsis* seedlings (for *AtDXS2*, no transcripts were detected; see Fig. S6). We could confirm that DXS protein was significantly increased 6 hrs after fosmidomycin treatment. For both isoforms, transcripts transiently increased only after 24 hrs, confirming the notion that the early increase of DXS protein was not due to transcriptional up-regulation (Fig. 7A,B). Thus, comparing the response of *C. roseus* young leaves and *Arabidopsis* seedlings (cotyledon stage) to fosmidomycin treatment, the results indicate that in both species the observed up-regulation of DXS protein is uncoupled from the level of DXS transcripts.

### Cycloheximide strongly inhibits fosmidomycin-induced accumulation of DXS protein

**Figure 8:**
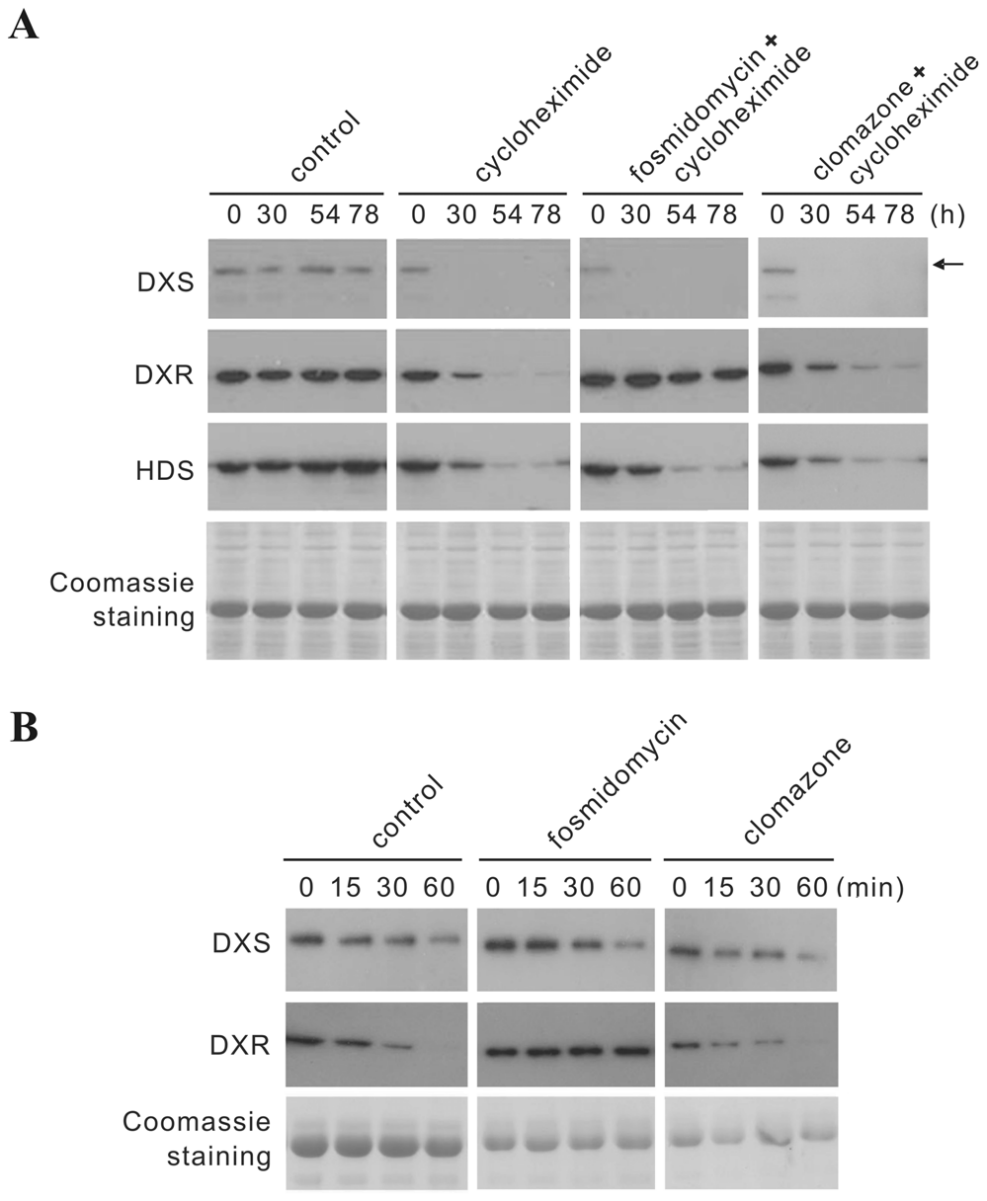
Time course of *in vivo* cycloheximide treatment as compared to simultaneous treatment with fosmidomycin or clomazone, respectively, and subsequent turnover of DXS and DXR proteins in isolated chloroplasts. **(A)** for each plant, two pairs of mature leaves were injected with 100 µM cycloheximide solution alone, or in combination with 50 µM fosmidomycin or 50 µM clomazone, respectively. For each time point, young leaves were collected from three independent plants and processed for immunoblot analysis of DXS, DXR and HDS, respectively. **(B)** chloroplasts from young leaves of 6-week-old soil-grown *C. rosues* plants, treated with 50 µM fosmidomycin, 50 µM clomazone, or water (control) were isolated 78 hours after leaf injection (see (A) this Fig.). Chloroplasts were incubated for 1 h in the light (100 µmol m^−2^ s^−1^) at 25°C in the presence of 5 mM ATP and fosmidomycin or clomazone (50 µM). Aliquots were taken at 0, 15, 30 and 60 minutes and used for protein extraction. DXS and DXR proteins were detected by immunoblot. To obtain similar signal intensity at time point 0, the loading amount of protein from control samples (chloroplast isolated from water infiltrated plants) was twice that of fosmidomycin or clomazone samples (see also Fig. 5C).

The observed accumulation of DXS protein in response to fosmidomycin treatment is apparently the result of posttranscriptional control(s). Possible mechanisms could involve increased synthesis (e.g. by increased polysome-recruitment of *CrDXS2A&B* transcripts) and/or increased protein stability due to slowing protein turnover. To further explore possible mechanism(s), we blocked cytosolic translation with cycloheximide. A 100 µM cycloheximide solution was applied either alone or in combination with 50 µM fosmidomycin or clomazone as described above. Tissue samples from young leaves were taken at 0 to 78 hrs after inhibitor application and analyzed for MEP pathway enzyme expression at protein level (Fig. 8A). Note that application of cycloheximide resulted in slight wilting and anthocyanin accumulation (“violet phenotype”, not shown), and when applied together with fosmidomycin or clomazone, cycloheximide retarded leaf development and prevented the fosmidomycin or clomazone-associated bleaching phenotype.

Treatment with cycloheximide alone caused a rapid decline of DXS, DXR and HDS proteins, revealing not only an efficient block of translation but also indicating high protein turnover. When applying cycloheximide together with fosmidomycin, the decline of DXS and HDS proteins followed a similar time course, indicating that the strong accumulation of DXS protein after fosmidomycin treatment results from increased *de novo* protein synthesis, presumably by increased polysome-recruitment of *CrDXS2A&B* transcripts (see above).

Unexpectedly, DXR protein level remained relatively stable when fosmidomycin and cycloheximide were applied simultaneously (Fig. 8A). Thus, fosmidomycin apparently prevented the degradation of DXR protein observed upon cycloheximide treatment. Considering the mode of action of fosmidomycin [47], it may be speculated that its binding to DXR might change enzyme conformation, rendering it resistant to protein turnover via chloroplast-localized proteases (e.g. Clp protease). It is noteworthy that the presence of clomazone (which is converted *in planta* to the specific inhibitor 5-ketoclomazone, see above) did not protect DXS protein from turnover in the presence of cycloheximide (Fig. 8A).

### Fosmidomycin protects DXR enzyme from proteolytic turnover in the chloroplast

To confirm the hypothesis that fosmidomycin protects DXR protein from degradation, chloroplasts were isolated 78 hrs after treatment from control plants, fosmidomycin-treated plants and clomazone-treated plants, respectively. For the *in vitro* protein degradation experiment, chloroplasts were incubated for 1 h in the light at 25°C in the presence of 5 mM ATP (as described above). To compensate for fosmidomycin/clomazone loss/dilution during the chloroplast isolation procedure, the final incubation solution also contained 50 µM fosmidomycin/clomazone. Samples were taken at 0, 15, 30, 60 min and analyzed by immunoblotting for DXS and DXR protein, respectively (Fig. 8B). While turnover of DXS was not affected by fosmidomycin or clomazone (for comparison see also Fig. 5C), DXR protein was clearly stabilized in the presence of fosmidomycin, supporting the hypothesis that specific binding of fosmidomycin to DXR protein protects the latter against degradation via plastidic protease(s).

## Discussion

The molecular basis for higher plant MEP pathway regulation at different levels has been the focus of several recent studies [17], [20], [23], [25], [26]. As this chloroplast-localized but nuclear-encoded pathway delivers not only precursors for photosynthetic components (chlorophylls, carotiniods), plant hormones (abscisic acid, giberellic acid) and antioxidants (tocopherols), but also for monoterpenoids and derived secondary metabolites of high pharmacological value [8], [48], understanding the different modes of its regulation is of fundamental importance for basic and applied research.

Hitherto, molecular studies have largely focused on the model plant *Arabidopsis*, unraveling several aspects of posttranscriptional regulation of MEP pathway enzyme expression [23], [25], [26], and or on the differential regulation of DXS isoforms and their specific roles in housekeeping functions for photosynthesis (i.e. type I) and stress-related functions (i.e. type II), respectively, with a major focus on transcriptional control [15], [45], [49], [50]. With the present study, we provide evidence for both transcriptional and posttranscriptional controls affecting the expression of DXS and DXR, respectively, in the pharmacologically important plant species *C. roseus*.

As an important initial step of the present study, a type I DXS isoform was cloned from *C. roseus* (Fig. S1). Since comprehensive analysis of all primary RT-PCR products did not deliver any related type I DXS sequence, the cloned *CrDXS1* cDNA apparently represented the only housekeeping leaf-expressed DXS isoform. For further confirmation of its housekeeping function, we compared the expression of all three *CrDXS* isoforms in different plant tissues (Fig. 2) and in leaf discs exposed to oxidative stress via paraquat treatment (Fig. 3). The results reveal that *CrDXS1* transcripts are prevalent in young and mature leaves, however with lower *CrDSX2A&B* mRNA levels also being detected in young leaves, whereas roots exclusively express *CrDXS2A&B* transcripts (Fig. 2), in agreement with expression patterns observed in other dicot and monocot species [15],[45]. Furthermore, in leaf tissue oxidative stress induces only *CrDXS2A&B* transcript accumulation, whereas *CrDXS1* mRNA declines (Fig. 3); similar observations were reported for plants under biotic and abiotic stress conditions [12], [15], [44], [50]. Note that in most plant species the consensus thiamine pyrophosphate (TPP)-binding domain, a highly conserved asparagine (N236 in CrDXS2A) residue in type II DXS isoforms is replaced by aspartic acid in type I DXS isoforms (Fig. S2). Whether this substitution is of functional significance remains to be shown.

Interpretation of the oxidative stress experiment (Fig. 3) has to acknowledge that leaf disc preparation causes a wound response also in the “control” treatment, thus the comparison is between wounding and wounding with simultaneous chloroplast-derived oxidative stress. Interestingly, wounding as such did not cause significant changes of MEP pathway gene transcripts, and, likewise, MEP pathway protein levels remained rather stable, in marked contrast to additional oxidative stress, which strongly but differentially affected protein levels of CrDXS, DXR and HDS (Fig. 3). Rivasseau et al. [22] demonstrated that in spinach leaves oxidative stress damaged HDS, an O_2_-hypersentitive [Fe_4_-S_4_]-protein, providing a possible explanation for the massive loss in HDS protein (Fig. 3). In line with an important role of MEP pathway imbalance for stress signaling, Xiao et al. [51] have demonstrated that abiotic stress causes an increase in the HDS substrate methylerythritol cyclodiphosphate (MEcPP), which in turn acts as retrograde signal inducing the expression of nuclear-encoded stress genes.

Previously, it has been shown that *CrDXS* expression was stimulated in ORCA3 overexpression *C. roseus* cell lines [34] and plants [35], however, those reports addressed only the *CrDXS2A* isoform. Here, we have compared the response of all three DXS promoter sequences (*CrDXS1p, CrDXS2Ap&Bp*) to the transcription factor ORCA3, using the dual luciferase assay for reporting *in vivo* promoter activities. The results indicate that only type II DXS isoforms are regulated by ORCA3. The only moderate induction of DXS by ORCA3 in leaf tissue, which is known to synthesize MIAs [35], suggests that other transcription factors may be involved in the regulation of DXS.

Having demonstrated the differential regulation of DXS isoforms in *C. roseus* in response to ORCA3 and chloroplast-derived oxidative stress, we have used this information to study the expression of MEP pathway enzymes in response to selective inhibition of MEP pathway flux at DXS and DXR level, respectively (Fig 5s. -Fig 6, Fig 7, Fig 8). Using MEP pathway efficient inhibitors 5-ketoclomazone (or its immediate precursor clomazone) and fosmidomycin, an efficient *in vivo* application technique (which is non-invasive for the targeted tissue, i.e. young leaves) has been established. The method for *in vivo* delivery of the DXS and DXR inhibitors (and cycloheximide, see below) via remote injection of inhibitor to fully expanded leaves relies on the fact that the injected compounds are phloem-mobile [52], [53] and are therefore rapidly transported with the assimilation stream to the shoot apex and young “sink” leaves. Mock leaf infiltration with deionized water did not cause any visible phenotype in the young leaves, nor did it induce changes in gene expression. Thus, the chosen protocol appears to be a highly efficient method for non-invasive (*in planta*) inhibitor delivery. While clomazone blocks the formation of 1-deoxy-D-xylolose 5-phosphate (DXP) and thus also prevents precursor flux towards thiamine biosynthesis [54], fosmidomycin allows the accumulation of DXP but interrupts the downstream MEP pathway. Note that for DXR of *Plasmodium falciparum*, the structure of a fosmidomycin-DXR complex has already been solved, indicating tight binding of fosmidomycin to the active site of DXR enzyme [47]. While the exact binding mechanism of 5-ketoclomazone to DXS remains to be elucidated, a recent study on DXS from *Haemophilus influenzae* has demonstrated that the inhibitor's binding site differed from the binding sites for DXS enzyme substrates pyruvate and D-glyceraldehyde 3-phosphate, respectively [55].

The first important conclusion from *in vivo* treatments with clomazone/5-ketoclomazone (Figs. 5A, S6A) and fosmidomycin (Fig. 6A) is that, like in *Arabidopsis* and maize seedlings [15], [23], [46], the amount of DXS protein is increased, *C. roseus* showing a particularly strong response. However, while in *Arabidopsis* and maize, the increase of DXS protein in response to both inhibitors occurred in the absence of any change in DXS transcript amount, accumulation of DXS protein in *C. roseus* in response to clomazone correlated with a moderate increase of *CrDXS2A&B* transcripts (Fig. 5); in contrast, fosmidomycin treatment caused only a slight and transient increase of *CrDXS2A&B* mRNAs (Fig. 6B). Interestingly, in *Arabidopsis* the fosmidomycin-induced DXS protein increase is due to accumulation of AtDXS1 protein ( = AtCLA1), which belongs to clade I (see Fig. S1), consistent with the induction of AtCLA1 protein in response to clomazone (Kobayashi et al., [46]). It is noteworthy that, although containing 3 DXS genes in its genome, *Arabidopsis* appears to be exceptional with respect to the absence of a type II DXS [49].

The stability of CrDXS protein in isolated chloroplasts of clomazone-exposed leaves was not increased (but rather reduced (Fig. 5C)), supporting the notion that the protein accumulation observed *in vivo* is the result of de *novo* synthesis. Whether the increase in *CrDXS2A&B* mRNAs as such fully accounts for the massive CrDXS protein accumulation, or if increased translation efficiency is also involved remains to be shown. The observation that blocking MEP pathway flux at either DXS or DXR enzyme level caused a comparable accumulation of CrDXS protein, while only for clomazone treatment *CrDXS2A&B* transcripts showed a substantial increase (in contrast to fosmidomycin treatment) argues in favor of a common mechanism (at least partially) independent of transcript increase. For other stress-factors, e.g. anaerobiosis, substantial transcript unloading and *de novo* recruitment of stress-induced transcripts strongly contributes to reprogramming gene expression [56]. Thus, *CrDXS2A&B* transcripts may have been preferentially recruited to polysomes under stress conditions.

To address the *in vivo* turnover of MEP pathway enzymes, the same experimental setup used for DXS and DXR inhibitors (see above) was used to deliver cycloheximide to the young developing leaves (Fig. 8A). For an incubation of up to 78 hrs, plants showed only mild visible stress syndromes (slight wilting; visible anthocyanin accumulation), but further shoot growth was retarded. Unexpectedly, analysis of protein expression levels revealed that simultaneous application of fosmidomycin and cycloheximide protected DXR protein from turnover, this effect being specific for DXR when compared with turnover of DXS and HDS protein, respectively. As this apparent protection of DXR from degradation could be confirmed in isolated chloroplasts (Fig. 8B), we hypothesize that the substrate-like tight binding of fosmidomycin to the active site of DXR [47] either shields protease-sensitive sites or causes a conformational change of DXR rendering it resistant to chloroplast protease(s), e.g. Clp protease [26], [32]. Thus, fosmidomycin inhibits but also stabilizes the DXR enzyme. The apparent absence of a fosmidomycin-mediated stabilization and subsequent accumulation of DXR protein *in vivo* (Fig. 6A) may be due to the fact that in intact cells (control treatment) it is the substrate DXP which stabilizes the DXR protein, whereas in isolated chloroplasts the supply of DXP is interrupted. Regarding the observed substantial turnover of MEP pathway enzymes *in vivo* (Fig. 8A) and in isolated chloroplasts (Figs. 5C&8B), it is tempting to speculate that *in vivo* the level of enzyme substrates may impact on the stability of MEP pathway enzymes. Recent proteomic studies have confirmed that impaired Clp-protease function causes accumulation of MEP pathway enzyme proteins, e.g. DXR and HDS [32].

In summary, the present study provides new insight into the regulation of MEP pathway gene expression, in particular into mechanisms affecting the steady state levels of the MEP pathway enzymes DXS and DXR in a plant species of high pharmaceutical importance. Clearly, posttranscriptional mechanisms are involved which are likely to include increased protein synthesis (e.g. polysome recruitment) and modulation of protein turnover by chloroplast proteases (e.g Clp protease). As the biosynthesis of MIAs in *C. roseus* is highly regulated and involves several cell types [57], [58], [59], the novel non-invasive protocol for inhibitor delivery will allow to explore the *in planta* consequences of changed MEP pathway flux for overall MIA accumulation. In particular, the localization of *CrDXS2A* mRNA in internal phloem parenchyma cells [38] and the pronounced differential expression of CrDXS isoforms in response to MEP pathway perturbation (this study) may reflect the plant's metabolic strategy to redirect MEP pathway flux under stress exposure and in response to developmental cues.

## Materials and Methods

### *C. roseus* plant cultivation and chemical treatment procedures

Madagascar periwinkle (*Catharanthus roseus* [L.] G. Don, Apocynaceae) plants were grown in a growth chamber at 25°C with 14 hrs light period (fluorescent white light, 170 µmol m^−2^ s^−1^), and 22°C in the dark. If not indicated otherwise, 6-week-old plants (i.e. before onset of flowering) were used for chemical treatments.

For oxidative stress experiments, leaf discs (diameter 5 mm) were collected from fully expanded leaves, pooled on deionized water, and subsequently distributed to petri dishes to float on deionized water (for control) or 0.5 µM paraquat solution. Leaf discs were allowed to equilibrate with this solution in the dark for 1 h, and were thereafter transferred to continuous light in a SANYO growth cabinet at 25°C with continuous fluorescent white light (270 µmol m^−2^ s^−1^). Leaf discs were harvested at 0, 10, 16, 24, 30 hrs after treatment and further processed for protein and RNA isolation.

For inhibitor experiments (i.e. 50 µM clomazone, Sigma; 50 µM fosmidomycin, Invitrogen; 50 µM 5-ketoclomazone, a gift from K. Grossmann, BASF; 100 µM cycloheximide, Sigma; all in aqueous solution), inhibitor solution (approx. 1 ml total) was applied to the lower epidermis of each of the first two pairs of fully expanded leaves via a 1 ml needleless syringe; control plants were injected with deionized water. Note that based on initial screening experiments with different inhibitor dosage, inhibitor concentrations were chosen such as to cause a visible phenotype after 48 hrs. Injection of inhibitor solution was stopped when the solution had visibly penetrated the entire leaf. Per sampling point, young leaves of two to three independent plants were collected at 0, 4, 10, 30, 54, 78 and 102 hrs after chemical treatment. The pooled plant material was homogenized with a Retsch MM301 mill under liquid nitrogen and stored at −80°C until further use.

### RNA isolation and cDNA synthesis

Total RNA was extracted from frozen and homogenized tissue with the GeneMATRIX Universial RNA purification Kit (Roboklon, Berlin, Germany), according to the manufacturer's instructions. RNA quality was verified by agarose gel electrophoresis in addition to the absorbance ratios A_260_/A_280_. For qRT-PCR experiments, first strand cDNA synthesis was performed immediately after DNase (AppliChem, Germany) treatment by using AMV-Reverse Transcriptase (Roboklon, Berlin, Germany), following the manufacturer's instructions. For RACE cDNA template production, two parallel cDNA synthesis steps were carried out on 2 µg of total RNA extracted from *C. roseus* leaves. The cDNA for 5′-RACE was synthesized using a modified lock-docking oligo (dT) primer and the SMART II A oligo, whereas the 3′-RACE cDNA was synthesized using a traditional reverse transcription procedure, but with a special oligo (dT) primer provided with the kit. For details see the user manual of Smart™ Race cDNA amplification kit (Clontech, Germany).

### Cloning of *CrDXS1* full-length cDNA by RACE (Acc. No. KC625536)

Degenerate oligonucleotides were derived from conserved motives in type I *DXS* sequences from *Capsicum annuum* (Y15782), *Lycopersicon esculentum* (AAD38941), *Nicotiana tabacum* (CBA12009) and *Arabidopsis thaliana CLA1* (U27099). The forward primer *DXSI* FW: 5′- CCHATGCATSARYTKGCNGCDAAAGTDGATG -3′ and the reverse primer *DXSI* Rev: 5′- GCCTTCACAVGCYARNCCHGCAGC -3′ were derived from the amino acid sequences PMHELAAKVD and AAGLACEG, respectively. This primer pair was used to amplify a core fragment of 504 bp by standard gradient (from 48°C to 58°C) PCR amplification from *C. roseus* leaf cDNA. After gel purification, the PCR product was cloned into the pGEM-T vector (Promega) and sequenced. The sequence confirmed fragment was subsequently used to design gene specific primers for the cloning of the full-length cDNA of *CrDXSI* by RACE according to Smart™ Race cDNA amplification kit (Clontech, Germany). The cDNA templates were synthesized as described above.

Primers used for each RACE PCR reactions were as follows: 5′RACE UPM (universal primer mix): CTAATACGACTCACTATAGGGCAAGCAGTGGTATCAACGCAGAGT; 5′RACE DXSI GSP (gene specific primer): CGGCTCCAACCAGTCCTGCCCTGTCC; 5′RACE nested UPM: AAGCAGTGGTATCAACGCAGAGT; 5′RACE DXSI nested GSP: GCCAGTCCCGCAGCAAAGGTTACAGCA; 3′RACE DXSI GSP: ATATTGTCGCTATTCATGCA; 3′-RACE Primer A: AAGCAGTGGTATCAACGCAGAGTAC. 5′- and 3′-RACE PCR reaction were performed using Jumpstart Taq DNA polymerase (Sigma). PCR conditions for 5′-RACE PCR were as follows: initial denaturation at 95°C for 4 min followed by 35 cycles of 95°C for 1 min, 68°C (annealing) for 1 min, 72°C (extension) for 2 min, and a single final incubation for 10 min at 72°C. This primary PCR product was diluted (1∶50) and used as template for the nested 5′-RACE PCR with 4 min initial denaturation at 95°C, followed by 35 cycles of 95°C for 1 min, 58°C for 1 min, 72°C for 2 min, and a single final incubation for 10 min at 72°C. PCR conditions for 3′-RACE were similar except that the annealing temperature was set at 50°C. Because the bands of 3′-RACE PCR products in the agarose gel were very sharp, no nested 3′ RACE PCR was performed. PCR products were agarose gel-purified (NucleoTrap® Gel Extraction Kit; Clontech, Germany), cloned into pGEM-T vector (Promega), and 6 independent clones were selected for sequencing. Contiguous sequences were checked for a continuous open reading frame (ORF) and homology with known plant type I DXS sequences. Sequences of the cloned 5′- and 3′-RACE products were assembled using the Vector NTI software (Invitrogen). Finally the sequence of the initially amplified *DXS1* fragment was joined to the 5′- and 3′-RACE sequences at the overlapping sites.

### Isolation of DXS promoter sequences

Seven blunt-end cutting restriction enzymes (*Eco*RV, *Dra*I, *Pvu*II, *Stu*I, *Nae*I, *Ssp*I and *Sca*I) were used individually to completely digest *C. roseus* genomic DNA. Each batch of digested genomic DNA was purified and ligated overnight at 16°C to the adaptors provided by the Universal GenomeWalker kit (Clontech, USA). Amplification of PCR products from DXS promoters by primary and secondary walk with appropriate primers (listed in Table S1) was performed according to the manufacturer's protocol.

### Plasmid construction for promoter analysis

A 2146 bp region of the *CrDXS1* promoter (Acc. No. KC625533; restricted with *Bam*HI and *Nco*I), a 2348 bp region of the *CrDXS2A* promoter (Acc. No. KC625534; restricted with *Not*I and *Sac*II), and a 2053 bp region of the *CrDXS2B* promoter (Acc. No. KC625535; restricted with *Bam*HI and *Nco*I), were cloned into the multiple cloning site (MCS) of pGreen II 0800-LUC. The open reading frame of ORCA3 (EU072424) was cloned into PB7GW2 by the Gateway technology (Invitrogen).

### *Agrobacteria*-mediated transformation of *C. roseus* leaves

Electrocompetent *Agrobacterium* C58C51 cells were transformed with empty pGreen II 0800-LUC vector, empty pB7GW2 vector, or pGreen II 0800-LUC, containing the respective DXS promoter sequence, or PB7WG2 vector containing ORCA3. Single colonies were inoculated into 5 ml of liquid YEB medium containing the appropriate selection antibiotics, and grown overnight at 28°C with vigorous shaking (180–200 rpm). 0.5 ml of the overnight culture were used to inoculate 50 ml of YEB medium including 5 µM acetosyringone and appropriate antibiotics, incubated at 28°C under vigorous shaking until reaching the stationary phase. Bacteria were harvested by centrifugation at 3,000*g* for 30 min at room temperature. Cells were resuspended in 10 ml of infiltration buffer (10 mM MgCl_2_, 10 mM MES (pH 5.8), 150 mM acetosyringone), supplemented with 10 µl L^−1^ Silwet L-77. Concentration of bacterial suspension was measured by spectroscopy and adjusted to OD_600_ 1.5 with infiltration buffer, incubated at room temperature for additional 2 hrs. Bacteria with DXS promoter constructs and ORCA3, respectively, were mixed at a 1∶1 ratio, and the resulting bacterial suspension was infiltrated into leaves via the lower epidermis with a needleless syringe.

### Dual-luciferase reporter assay

The dual-luciferase reporter assay was performed according to the procedure reported by Hellens and coauthours [43]. Approximately 10 mg of grinded leaf material was resuspended in 40 µl passive lysis buffer (PLB, Promega). Subsequently, *Firefly* and *Renilla* luciferase activities were determined using the Beetle and Renilla Glow Juice, containing the corresponding substrates (PJK, Kleinblittersdorf, Germany). Briefly, 20 µl of extract was mixed with 50 µl Beetle and Renilla Juice buffer in separate tubes and *Firefly/Renilla* activities were determined with the Lumat LB9507 Luminometer (Berthold Technologies). Relative luciferase activities were calculated as the ratio between the *Firefly* and the *Renilla* luciferase activities. All transfection experiments were performed four times.

### Protein extraction and immunoblotting

Total protein was extracted from frozen and homogenized plant materials (same starting material as used for quantitative real-time PCR, see below) in freshly prepared extraction buffer containing 10 mM Tris-HCl (pH 7.5), 0.1 mM EDTA, 0.1 mM MgCl_2_, 1 mM DTT, and 1 mM PMSF, followed by centrifugation for 30 min at 16.000 x g and 4°C. The protein concentration in the supernatant was determined with the Bradford reagent (Bio-Rad, Hercules, CA), using BSA as a standard. Protein samples were separated by SDS-PAGE on 11–12.5% polyacrylamide gels. Ten µg of protein was loaded per lane. After electrophoresis, proteins were transferred to a polyvinylidene difluoride (PVDF) membrane in a semi-dry blotting device (TransBlot SD, Bio-Rad). Immunodetection was performed with polyclonal antisera raised against recombinant proteins (full length ORFs of CrDXS2A, CrDXR and CrHDS, but without transit peptides) expressed in *E. coli*; all antisera were immunopurified using recombinant antigen protein. Specificity of the antisera was confirmed by i) agreement of immunosignal with predicted mature protein size, ii) absence of the immunosignal in the corresponding preimmune serum, and iii) successful suppression of immunosignal in the presence of excess recombinant antigen. An anti-rabbit inmunoglobulin horseradish peroxidase (HRP) conjugate was used as a secondary antibody (Pierce Biotechnology, Rockford, USA). Immunosignals were detected using the SuperSignal West Dura Extended Duration Substrate (Pierce Biotechnology, Rockford, USA).

### Transcript assessment by quantitative real time PCR (qPCR)

PCR reactions were performed in 96-well plates with the iCycler thermocycler (Biorad) using SYBR Green (S7563, Invitrogen) to monitor dsDNA synthesis. Each reaction contained a mixture of 5 µl diluted cDNA sample (equivalent to 15 ng RNA input), 0.5 µl of each primer (10 µM), 1.5 µl 10 × PCR buffer, 0.3 µl dNTPs (10 mM each), 0.15 µl Jumpstart Taq DNA polymerase (Sigma), 0.15 µl diluted (1∶400) SYBR Green, 0.6 µl diluted FITC (1∶4000, Bio-Rad) and water in a final volume of 15 µl. The gene-specific sets of primers are presented in Table S2 (*C. roseus*) and Table S3 (*A. thaliana*). The qRT-PCR reactions were carried out following the recommended thermal profile: 95°C for 5 min followed by 40 cycles of 95°C for 30 s, 58°C for 20 s and 72°C for 20 s. After 40 cycles, melting curves were determined by heating from 55°C to 95°C with a ramp speed of 0.5°C min^−1^. To determine primer efficiency, serial dilutions of the templates were conducted for all primer combinations. Each reaction was performed in triplicate, and the amplification products were examined by agarose gel electrophoresis and melting curve analysis. The expression stability of reference genes (*Actin*, *Ubiquitin*, *GAPDH* and *EF1α*) was assessed using geNorm algorithms, and the relative gene expression level was calculated by normalizing to the geometric mean of the reference genes according to a previously described method [60].

### Cultivation and fosmidomycin treatment of *Arabidopsis thaliana* seedlings

*A. thaliana* ecotype *Ler* seeds were germinated and grown under sterile conditions for 8 days (cotyledons fully expanded), and treated with 100 µM fosmidomycin solution as reported by [23].

### Chloroplast isolation and *in vitro* protein turnover experiments

Intact chloroplasts were isolated from young leaves of 6-week-old *C. roseus* plants, treated with clomazone or fosmidomycin via leaf infiltration (as described above), according to a previously described procedure [61]. Chloroplast protein degradation experiments were carried out according to the method described by Flores-Perez et al. [26]. In time course experiments, turnover of DXS and DXR proteins was monitored by separating proteins via SDS-PAGE, followed by immunoblot analysis as described above.

## Supporting Information

Figure S1 **Phylogenetic tree of plant DXS enzymes based on protein sequences, showing the separation into three clades.** Phylogenetic and molecular evolutionary analyses were conducted using MEGA version 4 [62]. The following plant sequences were included: CaTKT2 (*Capsicum annuum*, access number CAA75778), LeDXS (*Lycopersicon esculentum*, AAD38941), NtDXS (*Nicotiana tabacum*, CBA12009), EgDXS (*Elaeis guineensis*, AAS99588), CrDXS1 (*Catharanthus roseus*, KC625536), MtDXS1 (*Medicago truncatula*, CAD22530), AaDXS (*Artemisia annua*, AAD56390), AtCLA1 (*Arabidopsis thaliana*, AAC49368), AtDXS2 (*A. thaliana*, NP_850620), ApDXS1 (*Andrographis paniculata*, AAP14353), GbDXS1 (*Ginkgo biloba*, AAS89341), PaDXS1 (*Picea abies*, ABS50518), PdDXS1 (*Pinus densiflora*, ACC54557), OsDXS1 (*Oryza sativa*, NP_001055524), AtDXS3 (*A. thaliana*, NP_001078570), VvDXS1 (*Vitis vinifera*, XP_002277919), MpDXS (*Mentha piperita*, AAC33513), TeDXS (*Tagetes erecta*, AAG10432), CrDXS2B (*C. roseus*, ABI35993), PaDXS2A (*P. abies*, ABS50519), PdDXS2 (*P. densiflora*, ACC54554), GbDXS2 (*G. biloba*, AAR95699), PaDXS2B (*P. abies*, ABS50518), NpDXS (*Narcissus pseudonarcissus*, CAC08458), MtDXS2 (*M. truncatula*, CAD22531), SrDXS2 (*Stevia rebaudiana*, CAD22155), CrDXS2A (*C. roseus*, CAA09804) and LhDXS2 (*Lycopersicon hirsutum*, AAT97962), OsDXS2 (*O. sativa*, NP_001059086), VvDXS2A (*V. vinifera*, XP_002266925), VvDXS2B (*V. vinifera*, CBI17763), VvDXS2C (*V. vinifera*, XP_002270336), VvDXS2D (*V. vinifera*, XP_002271585), OsDXS3 (*O. sativa*, BAA83576), VvDXS3 (*V. vinifera*, XP_002282428). CrDXS isoforms are indicated with close circles. Bootstrap values are based on 1000 replications.
(DOCX)

Figure S2 **The transketolase consensus thiamine pyrophosphate (TPP) binding domain of DXS proteins.** The arrow marks the presence of negative charged amino acid aspartic acid (D) in type I DXS except for the gymnosperms *Picea abies*, *Pinus densiflora* and *Ginkgo biloba*, replaced by asparagin (N) in type II and III DXS.
(DOCX)

Figure S3 **Determination of DXS antiserum affinity to different DXS isoforms by immunoblot.** Purified His-tagged DXS1, 2A and 2B protein were subject to SDS-PAGE, transferred to the PVDF membrane, and detected by immunoblot with a polyclonal antiserum raised against recombinant CrDXS2A protein. The upper panel shows DXS specific immune signals. The lower panel indicates the loading of DXS1, 2A and 2B protein, respectively, at 500, 250, 125, 62.5 and 25 ng by silver staining.
(DOCX)

Figure S4 **Phenotypic changes (bleaching) in 0.5 µM paraquat-treated leaf discs from mature leaves of** ***C. roseus.*** Control discs did not show visible bleaching over 30 hrs, whereas bleaching starts in paraquat-treated discs after 16 hrs.
(DOCX)

Figure S5 **Phenotypic changes of 6-week-old** ***C. roseus*** **plants at 78 hrs after treatment with 50 µM clomazone or 50 µM fosmidomycin.** Clomazone or fosmidomycin solution was applied to the first two pairs of mature leaves with a 1 ml needleless syringe to the lower epidermis. Arrows mark the injected leaves. Note, that only developing leaves and still growing leaf tissues are bleached.
(DOCX)

Figure S6 **RT-PCR amplification products of** ***Arabidopsis*** **DXS isoforms separated on agarose.** Semi-quantitative RT-PCR amplification with isoform-specific primer sets. Note that *AtDXS2* transcripts level was extremely low in 8-day-old seedlings of *Arabidopsis thaliana*.
(DOCX)

Figure S7 **Effect of 5-ketoclomazone on expression of MEP pathway proteins and phenotypic changes in young leaves of 6-week-old** ***C. roseus*** **plants.** For each plant, the first two pairs of mature leaves were injected with a 50 μM 5-keto clomazone solution (or water for control) via a 1 ml needleless syringe to the lower epidermis. For each time point, young leaves from three independent plants were harvested and processed for MEP pathway protein analysis. **(A)** DXS, DXR and HDS proteins were detected by immunoblot with corresponding polyclonal antisera. **(B)** phenotypic changes in 5-keto clomazone and clomazone treated plants at 30, 54 and 78 hrs.
(DOCX)

Table S1 **Primers used for isolation of DXS promoters.**
(DOCX)

Table S2 **Primers for qPCR analysis in** ***C. roseus.***
(DOCX)

Table S3 **Primers for qPCR analysis in** ***A. thaliana.***
(DOCX)
