# Supplementary material for: Enzyme Inhibitor Studies Reveal Complex Control of Methyl-D-Erythritol 4-Phosphate (MEP) Pathway Enzyme Expression in Catharanthus roseus
Source: PLoS One. 2013 May 1;8(5):e62467. doi: 10.1371/journal.pone.0062467 (PMC3641079; doi:10.1371/journal.pone.0062467)
Supplement: Figure S1 — Phylogenetic tree of plant DXS enzymes based on protein sequences, showing the separation into three clades. Phylogenetic and molecular evolutionary analyses were conducted using MEGA version 4 [62]. The following plant sequences were included: CaTKT2 (Capsicum annuum, access number CAA75778), LeDXS (Lycopersicon esculentum, AAD38941), NtDXS (Nicotiana tabacum, CBA12009), EgDXS (Elaeis guineensis, AAS99588), CrDXS1 (Catharanthus roseus, KC625536), MtDXS1 (Medicago truncatula, CAD22530), AaDXS (Artemisia annua, AAD56390), AtCLA1 (Arabidopsis thaliana, AAC49368), AtDXS2 (A. thaliana, NP_850620), ApDXS1 (Andrographis paniculata, AAP14353), GbDXS1 (Ginkgo biloba, AAS89341), PaDXS1 (Picea abies, ABS50518), PdDXS1 (Pinus densiflora, ACC54557), OsDXS1 (Oryza sativa, NP_001055524), AtDXS3 (A. thaliana, NP_001078570), VvDXS1 (Vitis vinifera, XP_002277919), MpDXS (Mentha piperita, AAC33513), TeDXS (Tagetes erecta, AAG10432), CrDXS2B (C. roseus, ABI35993), PaDXS2A (P. abies, ABS50519), PdDXS2 (P. densiflora, ACC54554), GbDXS2 (G. biloba, AAR95699), PaDXS2B (P. abies, ABS50518), NpDXS (Narcissus pseudonarcissus, CAC08458), MtDXS2 (M. truncatula, CAD22531), SrDXS2 (Stevia rebaudiana, CAD22155), CrDXS2A (C. roseus, CAA09804) and LhDXS2 (Lycopersicon hirsutum, AAT97962), OsDXS2 (O. sativa, NP_001059086), VvDXS2A (V. vinifera, XP_002266925), VvDXS2B (V. vinifera, CBI17763), VvDXS2C (V. vinifera, XP_002270336), VvDXS2D (V. vinifera, XP_002271585), OsDXS3 (O. sativa, BAA83576), VvDXS3 (V. vinifera, XP_002282428). CrDXS isoforms are indicated with close circles. Bootstrap values are based on 1000 replications. (DOCX) [file pone.0062467.s001.docx]

**Supplementary Figure 1**


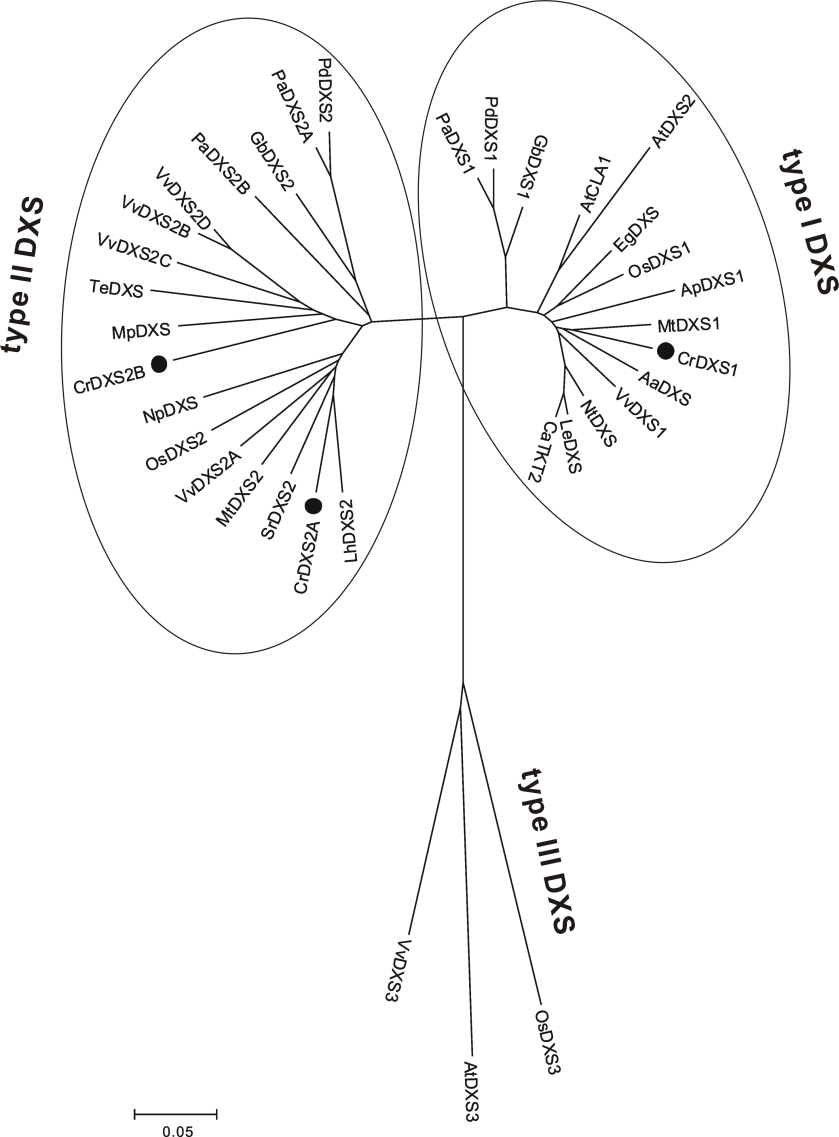


**Phylogenetic tree of plant DXS enzymes based on protein sequences, showing the separation into three clades**

Phylogenetic and molecular evolutionary analyses were conducted using MEGA version 4 (Tamura et al., [62]). The following plant sequences were included: CaTKT2 (*Capsicum annuum*, access number CAA75778), LeDXS (*Lycopersicon esculentum*, AAD38941), NtDXS (*Nicotiana tabacum*, CBA12009), EgDXS (*Elaeis guineensis*, AAS99588), CrDXS1 (*Catharanthus roseus*, KC625536), MtDXS1 (*Medicago truncatula*, CAD22530), AaDXS (*Artemisia annua*, AAD56390), AtCLA1 (*Arabidopsis thaliana*, AAC49368), AtDXS2 (*A. thaliana*, NP_850620), ApDXS1 (*Andrographis paniculata*, AAP14353), GbDXS1 (*Ginkgo biloba*, AAS89341), PaDXS1 (*Picea abies*, ABS50518), PdDXS1 (*Pinus densiflora*, ACC54557), OsDXS1 (*Oryza sativa*, NP_001055524), AtDXS3 (*A. thaliana*, NP_001078570), VvDXS1 (*Vitis vinifer*, XP_002277919), MpDXS (*Mentha piperita*, AAC33513), TeDXS (*Tagetes erecta*, AAG10432), CrDXS2B (*C. roseus*, ABI35993), PaDXS2A (*P. abies*, ABS50519), PdDXS2 (*P. densiflora*, ACC54554), GbDXS2 (*G. biloba*, AAR95699), PaDXS2B (*P. abies*, ABS50518), NpDXS (*Narcissus pseudonarcissus*, CAC08458), MtDXS2 (*M. truncatula*, CAD22531), SrDXS2 (*Stevia rebaudiana*, CAD22155), CrDXS2A (*C. roseus*, CAA09804) and LhDXS2 (*Lycopersicon hirsutum*, AAT97962), OsDXS2 (*O. sativa*, NP_001059086), VvDXS2A (*V. vinifer*, XP_002266925), VvDXS2B (*V. vinifer*, CBI17763), VvDXS2C (*V. vinifer*, XP_002270336), VvDXS2D (*V. vinifer*, XP_002271585), OsDXS3 (*O. sativa*, BAA83576), VvDXS3 (*V. vinifer*, XP_002282428). CrDXS isoforms are indicated with close circles. Bootstrap values are based on 1000 replications.
