# Supplementary material for: Enzyme Inhibitor Studies Reveal Complex Control of Methyl-D-Erythritol 4-Phosphate (MEP) Pathway Enzyme Expression in Catharanthus roseus
Source: PLoS One. 2013 May 1;8(5):e62467. doi: 10.1371/journal.pone.0062467 (PMC3641079; doi:10.1371/journal.pone.0062467)
Supplement: Figure S2 — The transketolase consensus thiamine pyrophosphate (TPP) binding domain of DXS proteins. The arrow marks the presence of negative charged amino acid aspartic acid (D) in type I DXS except for the gymnosperms Picea abies, Pinus densiflora and Ginkgo biloba, replaced by asparagin (N) in type II and III DXS. (DOCX) [file pone.0062467.s002.docx]

**Supplementary Figure 2**

**The transketolase consensus thiamine pyrophosphate (TPP) binding domain of DXS proteins**

The arrow marks the presence of negative charged amino acid aspartic acid (D) in type I DXS except for the gymnosperm *Picea abies*, *Pinus densiflora* and *Ginkgo biloba*, replaced by asparagine (N) in type II and III DXS.
