# Supplementary material for: Enzyme Inhibitor Studies Reveal Complex Control of Methyl-D-Erythritol 4-Phosphate (MEP) Pathway Enzyme Expression in Catharanthus roseus
Source: PLoS One. 2013 May 1;8(5):e62467. doi: 10.1371/journal.pone.0062467 (PMC3641079; doi:10.1371/journal.pone.0062467)
Supplement: Figure S3 — Determination of DXS antiserum affinity to different DXS isoforms by immunoblot. Purified His-tagged DXS1, 2A and 2B protein were subject to SDS-PAGE, transferred to the PVDF membrane, and detected by immunoblot with a polyclonal antiserum raised against recombinant CrDXS2A protein. The upper panel shows DXS specific immune signals. The lower panel indicates the loading of DXS1, 2A and 2B protein, respectively, at 500, 250, 125, 62.5 and 25 ng by silver staining. (DOCX) [file pone.0062467.s003.docx]

**Supplementary Figure 3**

**
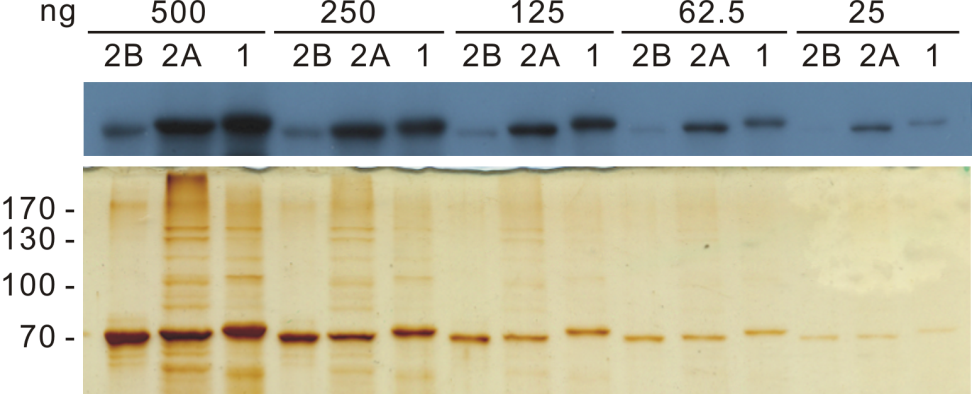
**

**Fig. S3**

**Determination of DXS antiserum affinity to different DXS isoforms by immunoblot**

Purified His-tagged DXS1, 2A and 2B protein were subject to SDS-PAGE, transferred to the PVDF membrane, and detected by immunoblot with a polyclonal antiserum raised against recombinant CrDXS2A protein. The upper panel shows DXS specific immune signals. The lower panel indicates the loading of DXS1, 2A and 2B protein, respectively, at 500, 250, 125, 62.5 and 25 ng by silver staining.
