# Supplementary material for: Enzyme Inhibitor Studies Reveal Complex Control of Methyl-D-Erythritol 4-Phosphate (MEP) Pathway Enzyme Expression in Catharanthus roseus
Source: PLoS One. 2013 May 1;8(5):e62467. doi: 10.1371/journal.pone.0062467 (PMC3641079; doi:10.1371/journal.pone.0062467)
Supplement: Figure S4 — Phenotypic changes (bleaching) in 0.5 µM paraquat-treated leaf discs from mature leaves of C. roseus. Control discs did not show visible bleaching over 30 hrs, whereas bleaching starts in paraquat-treated discs after 16 hrs. (DOCX) [file pone.0062467.s004.docx]

**Supplementary Figure 4**


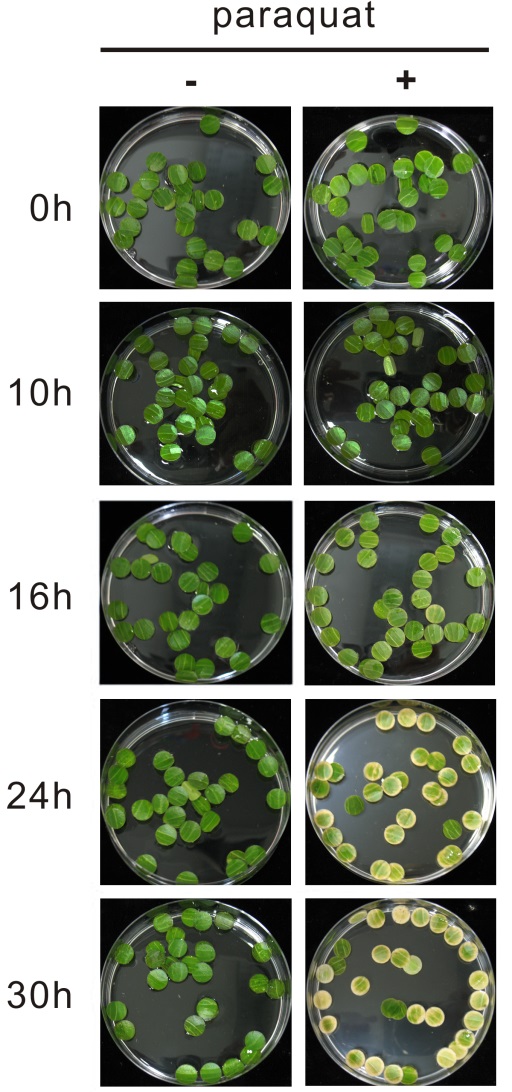


**Phenotypic changes (bleaching) in 0.5 µM paraquat-treated leaf discs from mature leaves of *C. roseus***

Control discs did not show visible bleaching over 30 hrs, whereas bleaching starts in paraquat-treated discs after 16 hrs.
