# Supplementary material for: Enzyme Inhibitor Studies Reveal Complex Control of Methyl-D-Erythritol 4-Phosphate (MEP) Pathway Enzyme Expression in Catharanthus roseus
Source: PLoS One. 2013 May 1;8(5):e62467. doi: 10.1371/journal.pone.0062467 (PMC3641079; doi:10.1371/journal.pone.0062467)
Supplement: Figure S5 — Phenotypic changes of 6-week-old C. roseus plants at 78 hrs after treatment with 50 µM clomazone or 50 µM fosmidomycin. Clomazone or fosmidomycin solution was applied to the first two pairs of mature leaves with a 1 ml needleless syringe to the lower epidermis. Arrows mark the injected leaves. Note, that only developing leaves and still growing leaf tissues are bleached. (DOCX) [file pone.0062467.s005.docx]

**Supplementary Figure 5**

A


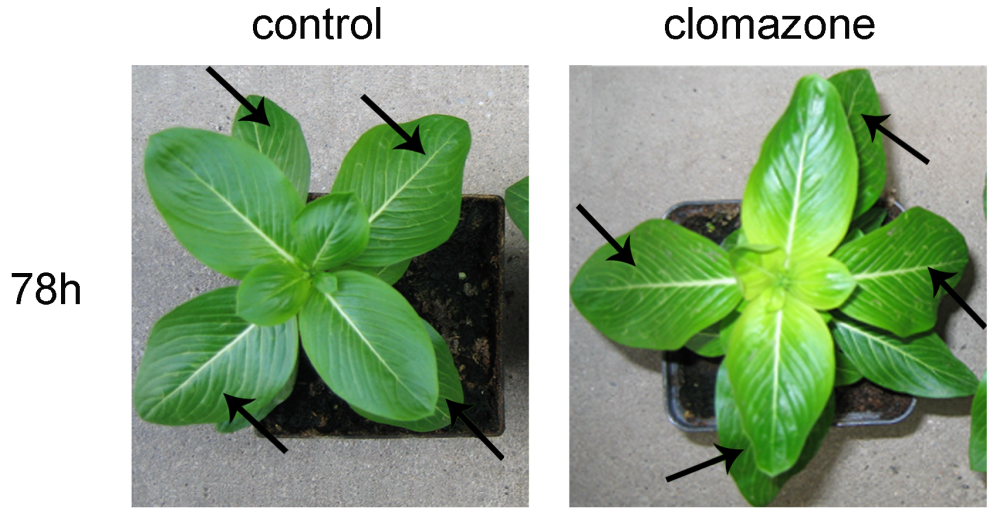


B


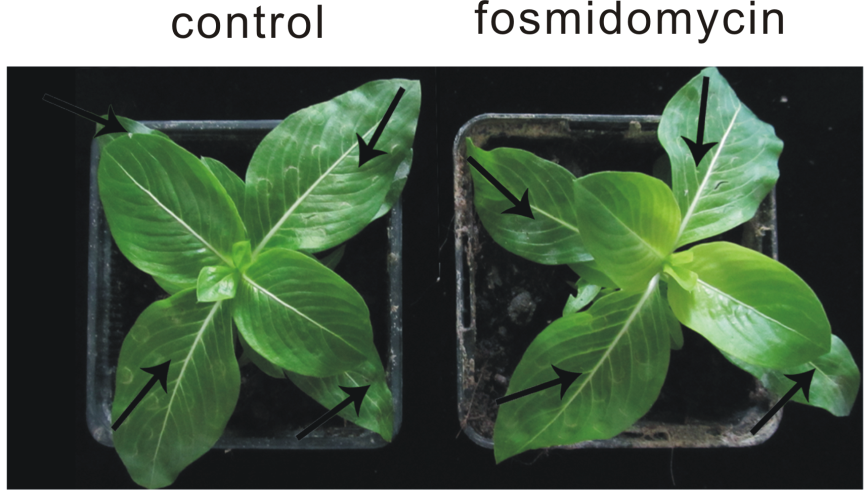


**78h**

**Phenotypic changes of 6-week-old *C. roseus* plants at 78 hrs after treatment with 50 µM clomazone (A) or 50 µM fosmidomycin (B)**

Clomazone or fosmidomycin solution was applied to the first two pairs of fully mature leaves with a 1 mL needless syringe to the lower epidermis. Arrows mark the injected leaves. Note, that only developing leaves and still growing leaf tissues are bleached.
