# Supplementary material for: Enzyme Inhibitor Studies Reveal Complex Control of Methyl-D-Erythritol 4-Phosphate (MEP) Pathway Enzyme Expression in Catharanthus roseus
Source: PLoS One. 2013 May 1;8(5):e62467. doi: 10.1371/journal.pone.0062467 (PMC3641079; doi:10.1371/journal.pone.0062467)
Supplement: Figure S6 — RT-PCR amplification products of Arabidopsis DXS isoforms separated on agarose. Semi-quantitative RT-PCR amplification with isoform-specific primer sets. Note that AtDXS2 transcripts level was extremely low in 8-day-old seedlings of Arabidopsis thaliana. (DOCX) [file pone.0062467.s006.docx]

**Supplementary Figure 6**


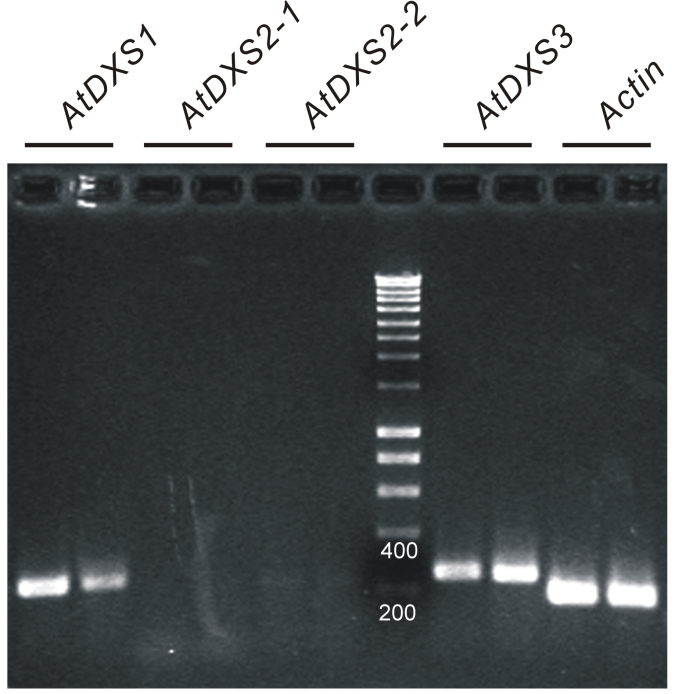


**RT-PCR amplification products of *Arabidopsis* DXS isoforms separated on agarose**

Semi-quantitative RT-PCR amplification with isoform-specific primer sets. Note that *AtDXS2* transcript level was extremely low in 8-day-old seedlings of *Arabidopsis thaliana*.
