# Supplementary material for: Enzyme Inhibitor Studies Reveal Complex Control of Methyl-D-Erythritol 4-Phosphate (MEP) Pathway Enzyme Expression in Catharanthus roseus
Source: PLoS One. 2013 May 1;8(5):e62467. doi: 10.1371/journal.pone.0062467 (PMC3641079; doi:10.1371/journal.pone.0062467)
Supplement: Figure S7 — Effect of 5-ketoclomazone on expression of MEP pathway proteins and phenotypic changes in young leaves of 6-week-old C. roseus plants. For each plant, the first two pairs of mature leaves were injected with a 50 μM 5-keto clomazone solution (or water for control) via a 1 ml needleless syringe to the lower epidermis. For each time point, young leaves from three independent plants were harvested and processed for MEP pathway protein analysis. (A) DXS, DXR and HDS proteins were detected by immunoblot with corresponding polyclonal antisera. (B) phenotypic changes in 5-keto clomazone and clomazone treated plants at 30, 54 and 78 hrs. (DOCX) [file pone.0062467.s007.docx]

**Supplementary Figure 7**

A


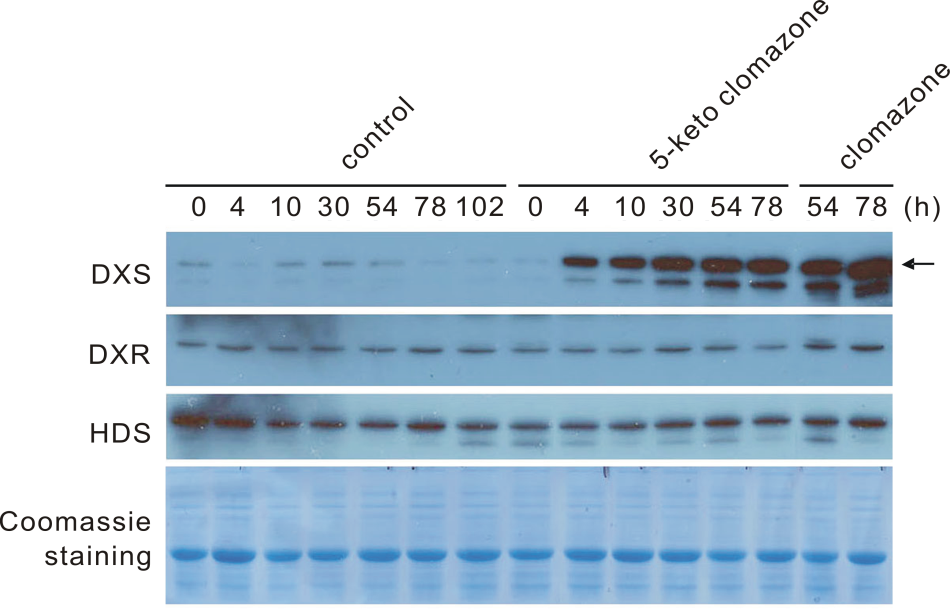


B


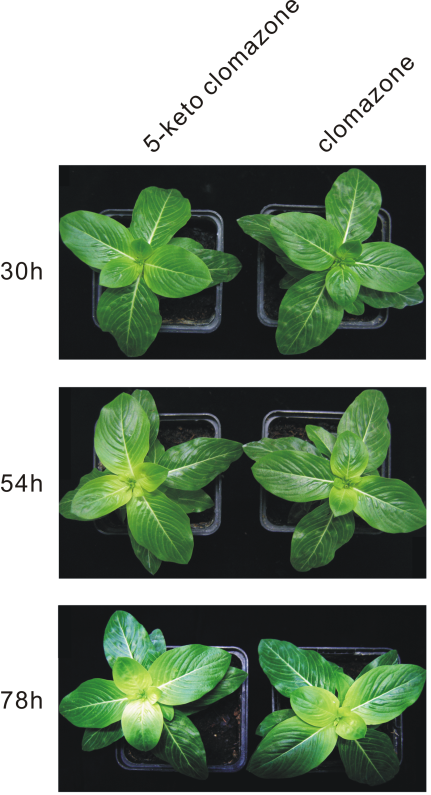


**Effect of 5-keto clomazone on expression of MEP pathway proteins and phenotypic changes in young leaves of 6-week-old *C. roseus* plants**

For each plant, the first two pairs of mature leaves were injected with a 50 μM 5-keto clomazone solution (or water for control) via a 1 ml needleless syringe to the lower epidermis. For each time point, young leaves from three independent plants were harvested and processed for MEP pathway protein analysis. (A), DXS, DXR and HDS proteins were detected by immunoblot with corresponding polyclonal antisera. (B), phenotypic changes in 5-keto clomazone and clomazone treated plants at 30, 54 and 78 hrs.
