# Supplementary material for: Enzyme Inhibitor Studies Reveal Complex Control of Methyl-D-Erythritol 4-Phosphate (MEP) Pathway Enzyme Expression in Catharanthus roseus
Source: PLoS One. 2013 May 1;8(5):e62467. doi: 10.1371/journal.pone.0062467 (PMC3641079; doi:10.1371/journal.pone.0062467)
Supplement: Table S1 — Primers used for isolation of DXS promoters. (DOCX) [file pone.0062467.s008.docx]

**Supplementary Table 1**.

Primers for isolation of DXS promoters

|  | **oligo name** | **sequence (5´- 3´), restricted sites underlined** |
| --- | --- | --- |
| Genomewalker kit primers | long adaptor: | GTAATACGACTCACTATAGGGCACGCGTGGTCGACGGCCCGGGCTGGT |
|  | short adaptor | PO4-ACCAGCCC-NH2 (5’ phosphate, 3’ aminolink C7, HPLC purified) |
|  | adaptor primer 1 | GTAATACGACTCACTATAGGGC |
|  | adaptor primer 2 | ACTATAGGGCACGCGTGGT |
| *DXS1* walker | DXSI GSP1 | TTCCGAAGCCACAGCCTTGTTCAAACTC |
|  | DXSI GSP2 | CTGGAAATGCAAATGCACAGAGAGCCATC |
|  | DXSI GSP3 | GGTCACTTCAAGAACAACACAAGTGGCAGTACC |
| *DXS2A* walker | DXS2A GSP1 | GGACTGTCCAATAAGCAGGAGAGATAGGCG |
|  | DXS2A GSP2 | ATGACAGCCCCGGAAACCGCCATGAATG |
| *DXS2B* walker | DXS2B GSP1 | CCATTCGATGCCACCGCCATTCTTTGAACC |
|  | DXS2B GSP2 | CTGGTTGGGTTGATGGAGCAGACTGATTGAG |
| *DXSI* promoter  (KC625533) | DXSI pro 5’BamHI | CGCGGATCC CGTATTATGCCCCTACATCC |
|  | DXSI pro3’ SacII | TCCCCGCGG CACAGATATATACCTCAATCTGTC |
| *DXS2A* promoter  (KC625534) | DXS2A pro 5’ BamHI | CGCGGATCC AAGAAATCTTGATAAGCTGTCC |
|  | DXS2A pro3’ SacII | TCCCCGCGG GAATGCAAAAGTGAATGAGAATTAG |
| *DXS2B* promoter  (KC625535) | DXS2B pro 5’BamHI | CGCGGATCC CTAGTATTAGTTTAATGACTCTCGAGC |
|  | DXS2B pro3’ SacII | TCCCCGCGG TCTTTGAACCTTTTTTTGGTTACC |
