# Supplementary material for: Enzyme Inhibitor Studies Reveal Complex Control of Methyl-D-Erythritol 4-Phosphate (MEP) Pathway Enzyme Expression in Catharanthus roseus
Source: PLoS One. 2013 May 1;8(5):e62467. doi: 10.1371/journal.pone.0062467 (PMC3641079; doi:10.1371/journal.pone.0062467)
Supplement: Table S2 — Primers for qPCR analysis in C. roseus. (DOCX) [file pone.0062467.s009.docx]

**Supplementary Table 2**

Primers for qPCR analysis in *C. roseus*

| **target genes** | **primer name** | **sequence (5´- 3´)** |
| --- | --- | --- |
| *CrDXS1* (KC625536) | DXS1_qFW | CGTGGGATGATTAGTGGTTC |
|  | DXS1_qREV | ATACTTGTCGGCTGCTCTCTC |
| *CrDXS2A* (AJ011840) | 2A_qFW | AGGTGAGATCCCTTTTTCTTCC |
|  | 2A_qREV | TTCTTGTGGCTTGCACATTTAG |
| *CrDXS2B* (DQ848672) | 2B_qFW | GGCTGGCCTAACTCCAAAG |
|  | 2B_qREV | TGATATTTTCCCCTAATTCCACA |
| *CrDXR* (AF250235) | DXR_qFW | TCAAGCAGAACTGGTAACTTCA |
|  | DXR_qREV | ACCAATACAAAGAAAACCCAACT |
| *CrHDS* (AY184810) | HDS_qFW | GGAGATGGAGTTATGTTGGAAG |
|  | HDS_qREV | AGGCAAATGTGATGTCTTTTCT |
| **reference genes** | **oligo name** | **sequence (5´- 3´)** |
| *Ubq* (FD415511) | UBIC_qFW | TCCTCCTTCCCTTCTCCA |
|  | UBIC_qREV | GGTTTGGGTCCGTCAACA |
| *GAPDH* (FD421703) | GAPDH_qFW | TCCTCTAATCTATCCCTGTAAAACC |
|  | GAPDH_qREV | TAGCCAAGGGAGCAAGACA |
| *EF1α* (EU007436) | EF1alpha_qFW | ATTGCCTTCGTCCCTATTTCT |
|  | EF1alpha_qREV | CCACCAATCTTGTAGACATCCT |
| *Actin* (FD417920) | Actin_qFW | GGCTGGATTTGCTGGAGATGAT |
|  | Actin_qREV | TAGATCCTCCGATCCAGACACTG |
| *RSP9* (AJ749993) | rsp_qFW | GAGGGCCAAAACAAACTTGA |
|  | rsp_qREV | CCCTTATGTGCCTTTGCCTA |
