# Supplementary material for: Enzyme Inhibitor Studies Reveal Complex Control of Methyl-D-Erythritol 4-Phosphate (MEP) Pathway Enzyme Expression in Catharanthus roseus
Source: PLoS One. 2013 May 1;8(5):e62467. doi: 10.1371/journal.pone.0062467 (PMC3641079; doi:10.1371/journal.pone.0062467)
Supplement: Table S3 — Primers for qPCR analysis in A. thaliana. (DOCX) [file pone.0062467.s010.docx]

**Supplementary Table 3**

Primers for qPCR analysis in *Arabidopsis*

| **target genes** | **primer name** | **sequence (5´- 3´)** |
| --- | --- | --- |
| *AtCLA1* (At4g15560) | AtDXS1_qFW | CGTTGTTCAGTTTCTTGCTCTC |
|  | AtDXS1_qREV | GGGTTGCATTTAGAGTGTTTGT |
| *AtDXPS2* (At3g21500) | AtDXS2_qFW | CCTTCTTGATGGAAAGCTCA |
|  | AtDXS2_qREV | ACAATGCTTCTCTAGGTGTTCC |
| *AtDXPS3* (At5g11380) | AtDXS3_qFW | GTGTTGGAGGGTTTGGTTCT |
|  | AtDXS3_qREV | CTCTTGTGAAGGTAAATCCCATA |
| **reference genes** | **primer name** | **sequence (5´- 3´)** |
| *AtUbi6* (NM_130278) | AtUbi6_qFW | GGTCTCACCTACGTTTACCAGA |
|  | AtUbi6_qREV | ATCCACAACATCCAAAAACAAC |
